# Supplementary material for: Propane wet reforming over PtSn nanoparticles on γ-Al2O3 for acetone synthesis
Source: Nat Commun. 2024 Sep 30;15:8470. doi: 10.1038/s41467-024-52702-x (PMC11443076; doi:10.1038/s41467-024-52702-x)
Supplement: Supplementary file 1 — Supplementary Information [file 41467_2024_52702_MOESM1_ESM.pdf]

Supplementary Information for

Propane wet reforming over PtSn nanoparticles on γ-Al_2O_3 for acetone synthesis

Xinlong Ma[1,2†], Haibin Yin[2†], Zhengtian Pu[2†], Xinyan Zhang[2], Sunpei Hu[2], Tao Zhou[2], Weizhe Gao[3], Laihao Luo[2]*, Hongliang Li[2,4*], Jie Zeng[1,2,5*]

[1]Deep Space Exploration Laboratory, Hefei, Anhui 230088, P. R. China

[2]Hefei National Research Center for Physical Sciences at the Microscale, University of Science and Technology of China, Hefei, Anhui 230026, P. R. China

[3]Department of Applied Chemistry, School of Engineering, University of Toyama, Gofuku 3190, Toyama 930-8555, Japan

[4]National Synchrotron Radiation Laboratory, University of Science and Technology of China, Hefei, Anhui, 230029 China

[5]School of Chemistry & Chemical Engineering, Anhui University of Technology, Ma'anshan, Anhui 243002, P. R. China

[†]These authors contributed equally to this work.

[*]e-mail: llh0214@ustc.edu.cn; lihl@ustc.edu.cn; zengj@ustc.edu.cn.

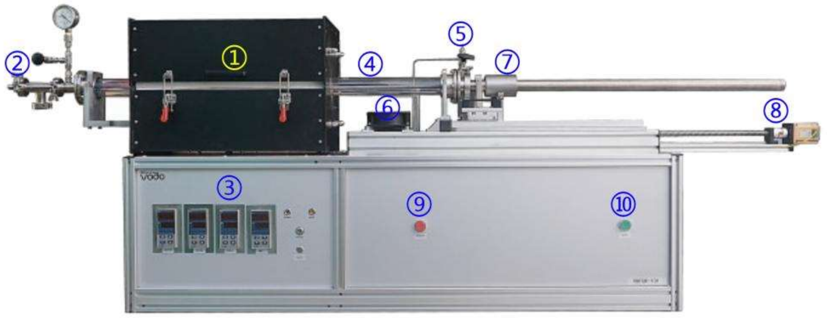

Supplementary Figure 1 | Modified tube furnace. ① Heating furnace; ② Vacuum system; ③ Electronic operating interface; ④ Quartz tube; ⑤ Gas pipelines; ⑥ Cooling system; ⑦ Magnetic lever system; ⑧ Motor control system; ⑨ Injection push button; ⑩ Multi-function button (short press for the cooling position and long press for exiting).

Supplementary Table 1 | Pt and Sn contents of samples determined by ICP-AES.

Samples	Pt (wt%)	Sn (wt%)
γ-Al_2O_3	-	-
Pt/γ-Al_2O_3	2.84	-
PtSn/γ-Al_2O_3	2.71	1.73

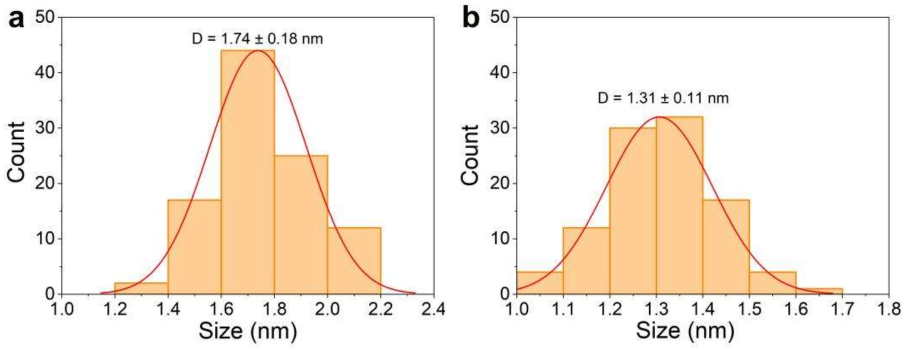

Supplementary Figure 2 | Size distribution. Size distribution of metal nanoparticles in (a) PtSn/γ-Al_2O_3 and (b) Pt/γ-Al_2O_3. Source data are provided as a Source Data file.

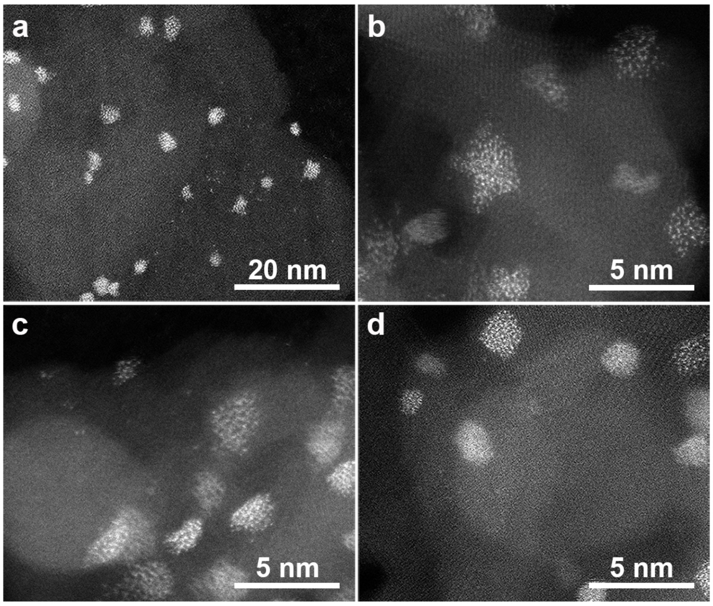

Supplementary Figure 3 | HAADF-STEM images of PtSn/γ-Al_2O_3. (a) HAADF-STEM image of PtSn/γ-Al_2O_3 at low magnification. (b-d) HAADF-STEM images of PtSn/γ-Al_2O_3 at high magnification.

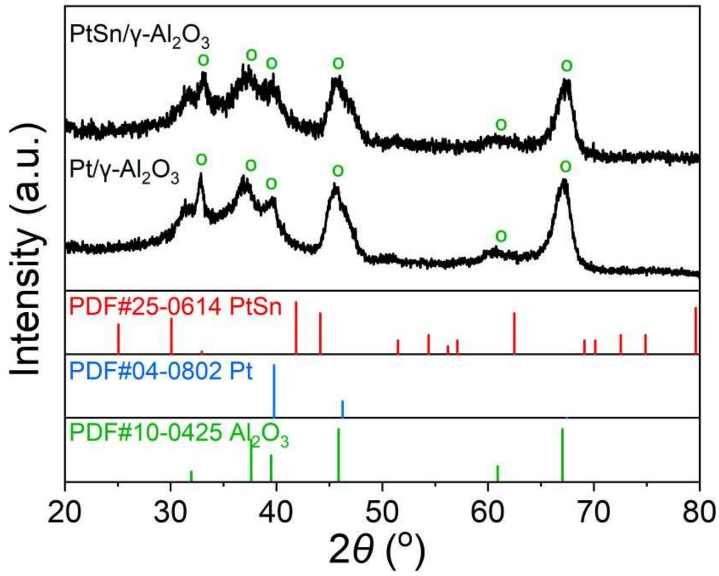

Supplementary Figure 4 | XRD patterns of PtSn/γ-Al_2O_3 and Pt/γ-Al_2O_3. Source data are provided as a Source Data file.

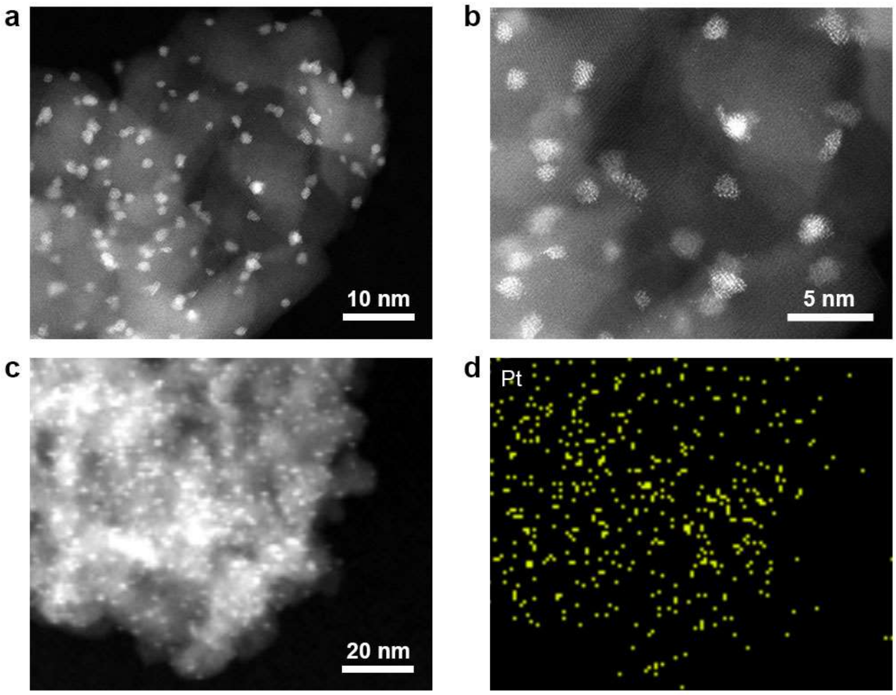

Supplementary Figure 5 | Characterizations of Pt/γ-Al_2O_3. (a, b) Magnified HAADF-STEM images of Pt/γ-Al_2O_3. (c) HAADF-STEM image and (d) corresponding EDS elemental mapping results for Pt/γ-Al_2O_3.

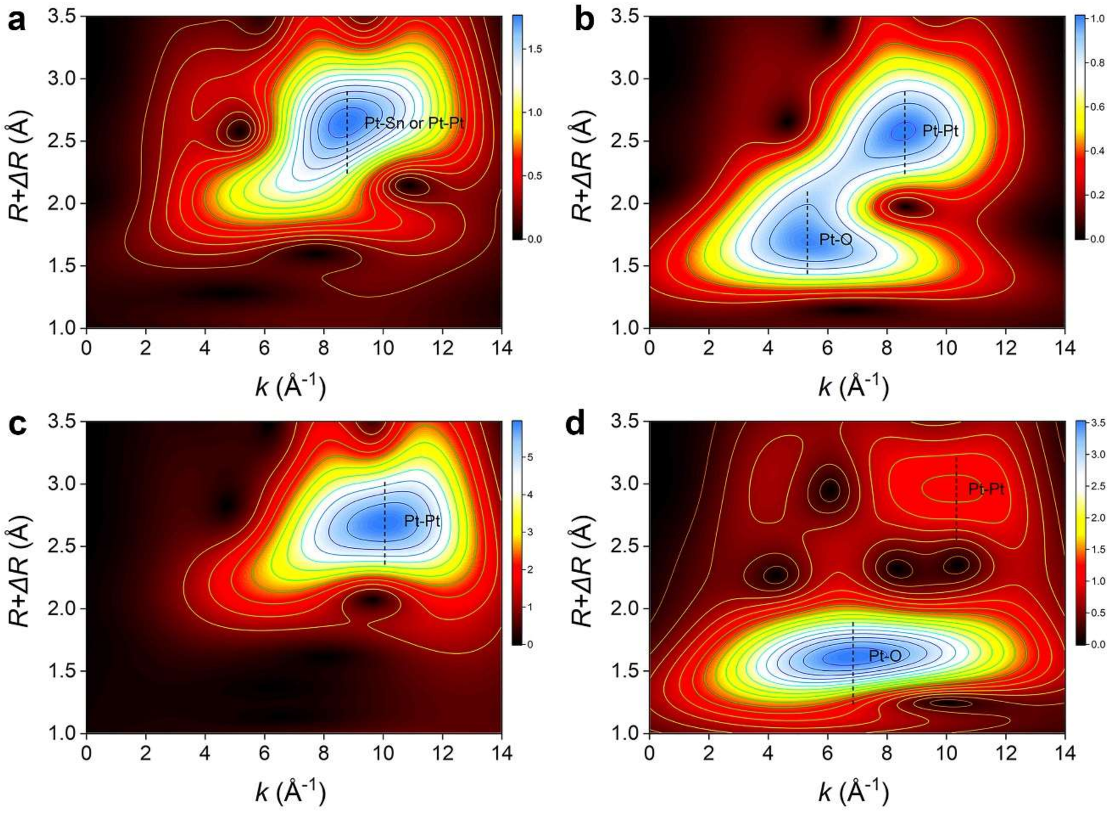

Supplementary Figure 6 | Wavelet transforms for the k^2-weighted EXAFS signals. (a) PtSn/γ-Al_2O_3, (b) Pt/γ-Al_2O_3, (c) Pt foil, and (d) PtO_2.

Notes:

In WT-EXAFS, the heavier the atom is, the higher *k* position the absorption center locates. The strongest absorption center in the WT-EXAFS spectrum of PtSn/γ-Al_2O_3 (panel **a**) was located at a lower K value compared with that for Pt-Pt bonds in panel b. As such, we used the combination of Pt-Sn and Pt-Pt coordination to fit the EXAFS results of PtSn/γ-Al_2O_3, though the bond length of Pt-Sn was similar to that of Pt-Pt. Source data are provided as a Source Data file.

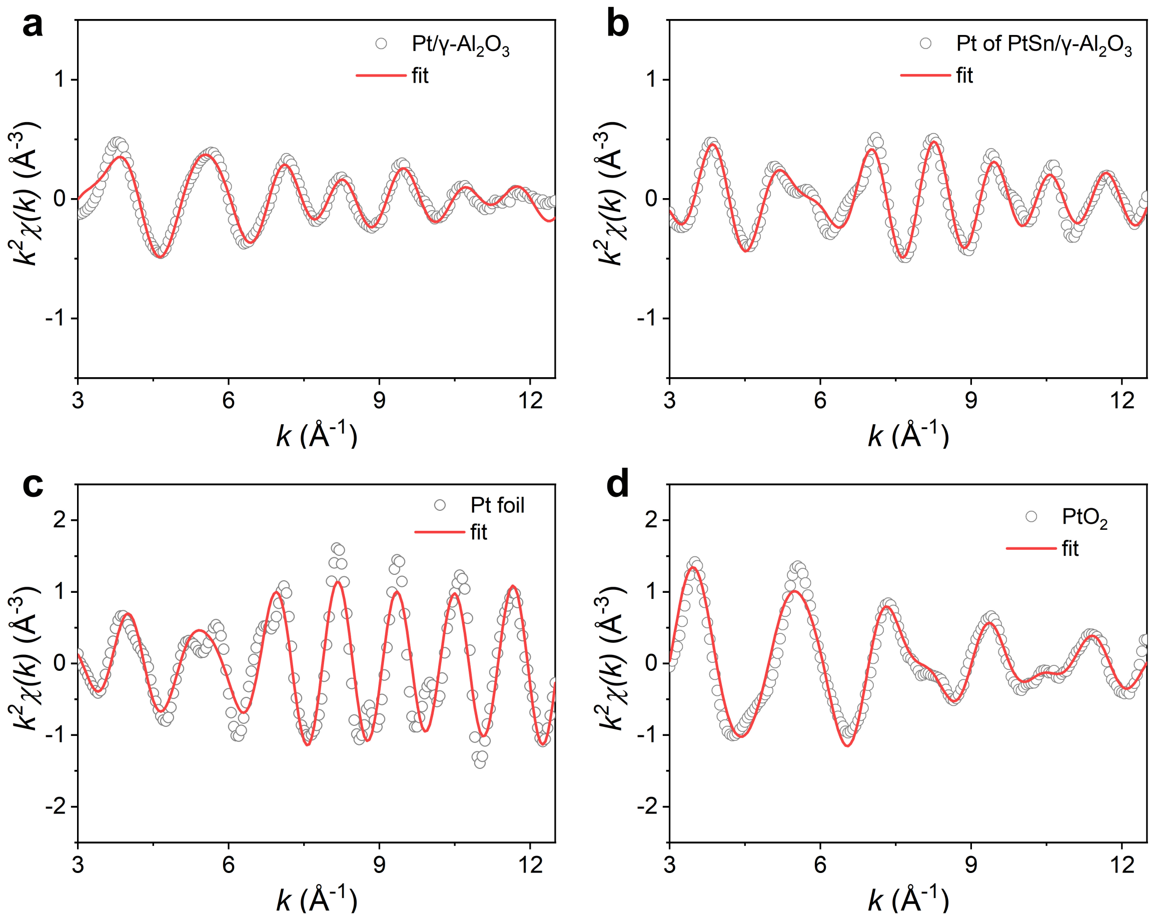

Supplementary Figure 7 | k^2-weighted experimental and fitting spectra. K^2-weighted experimental and fitting spectra with respect to Pt element in (a) Pt/γ-Al_2O_3, (b) PtSn/γ-Al_2O_3, (c) Pt foil and (d) PtO_2. Source data are provided as a Source Data file.

Supplementary Table 2 | EXAFS fitting parameters at the Pt L_3-edge for different samples.

Sample	Shell	N[a]	R(Å)[b]	σ^2(Å^2) [c]	ΔE_0 (eV) [d]	*R factor*
Pt foil	Pt-Pt	12.0	2.76±0.01	0.0044±0.0003	8.9±0.5	0.0005
PtO_2	Pt-O	6.0	1.99±0.01	0.0038±0.0001	3.1±1.5	0.0086
	Pt-Pt	6.0	3.11±0.11	0.0079±0.0021		
PtSn/γ-Al_2O_3	Pt-O	0.3±0.1	1.95±0.01	0.0155±0.0052	3.9±2.0	0.0020
	Pt-Sn	1.6±0.2	2.70±0.02	0.0064±0.0030		
	Pt-Pt	4.3±0.4	2.74±0.01	0.0013±0.0012		
Pt/γ-Al_2O_3	Pt-O	1.7±0.4	2.00±0.02	0.0107±0.0022	6.0±2.2	0.0056
	Pt-Pt	4.8±1.3	2.73±0.01	0.0091±0.0019		

[a] N: coordination numbers.

[b] R: bond distance.

[c] σ^2: Debye-Waller factors.

[d] ΔE_0: the inner potential correction. R factor: goodness of fit. S_0^2 was set to 0.776, according to the experimental EXAFS fit of Pt foil reference by fixing coordination numbers as the known crystallographic value.

We also attempted fitting without a Pt-Sn scattering path. Firstly, we replaced the Pt-Sn path with the Pt-Pt path, in other words, we have used two Pt-Pt paths and one Pt-O path for fitting. The R factor was 0.043, implying the poor matching degree. Then, we directly removed the Pt-Sn path, in other words, we have used one Pt-Pt path and one Pt-O path for fitting. The R factor reached 0.059, still implying the poor matching degree. Therefore, the Pt-Sn should be taken into consideration. We attribute the large Debye-Waller factors (σ^2) to that the amorphous structure led to the increase in the disorder degree of the system[1, 2]. This phenomenon is consistent with our HAADF-STEM results (Fig. 2b and Supplementary Fig. 3) which proved the amorphous nature of the sample.

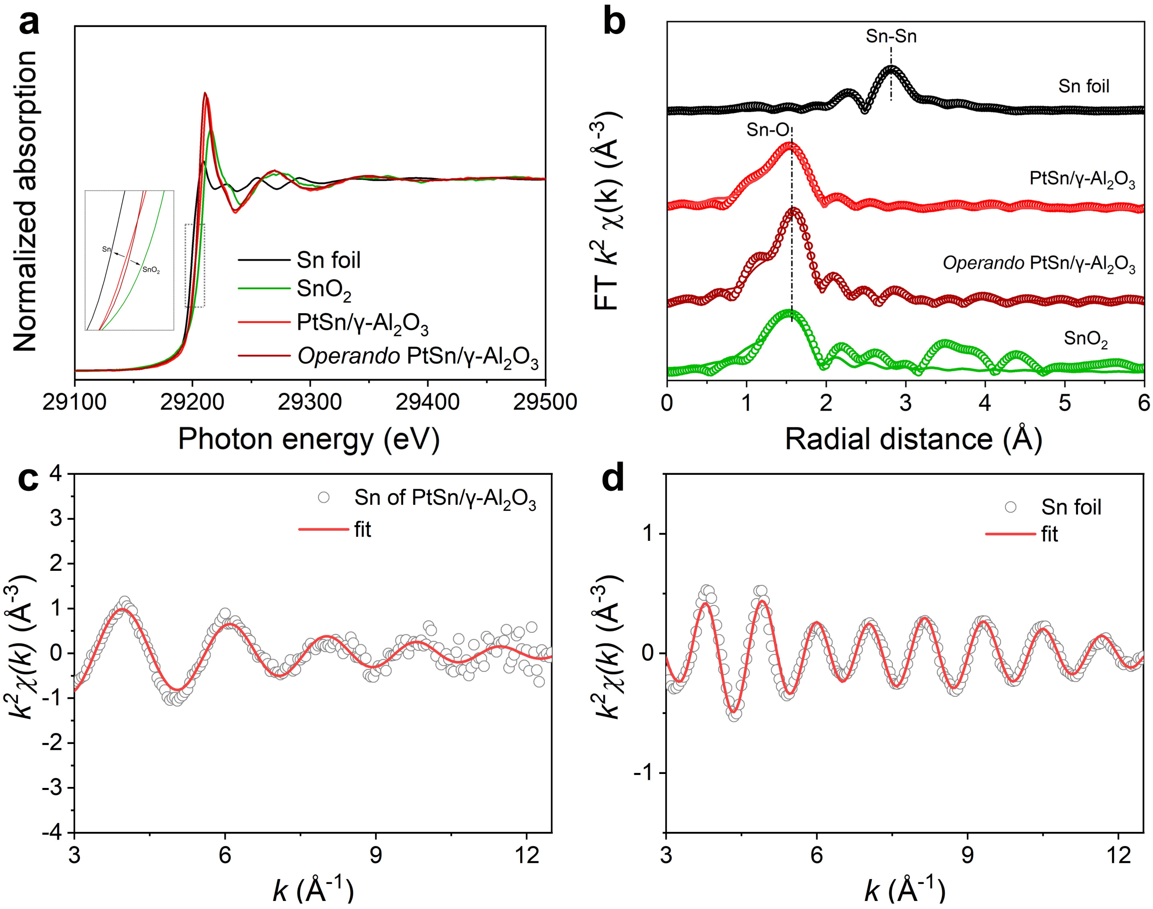

Supplementary Figure 8 | Sn K-edge XAFS spectra. (a) Sn K-edge XANES spectra of Sn foil, SnO_2, PtSn/γ-Al_2O_3 and operando PtSn/γ-Al_2O_3. (b) The corresponding Sn K-edge EXAFS spectra (circles) and curve fits (lines). *Operando* condition: 350 ºC, wet propane at 20 mL min^{-1}. The data are k^2-weighted and not phase-corrected. K^2-weighted experimental and fitting spectra with respect to Sn element in (c) PtSn/γ-Al_2O_3 and (d) Sn foil. Source data are provided as a Source Data file.

Supplementary Table 3 | EXAFS fitting parameters at the Sn K-edge for PtSn/γ-Al_2O_3.

Sample	Shell	N[a]	R(Å)[b]	σ^2(Å^2) [c]	ΔE_0 (eV)[d]	*R factor*[e]
PtSn/γ-Al_2O_3	Sn-O	5.0±0.5	2.01±0.02	0.0045±0.0035	-0.3±3.4	0.0048

[a] *N*: coordination numbers.

[b] *R*: bond distance.

[c] σ^2: Debye-Waller factors.

[d] ΔE_0: the inner potential correction.

[e] *R* factor: goodness of fit.

Supplementary Table 4 | Assignment of DRIFTS peaks in Figure 2f.

Catalyst	Surface species	Wavenumber (cm^{-1})	Reference
PtSn/γ-Al_2O_3	Linear Pt_{wc}-(CO)	2077	This work
	Linear Pt_{uc}-(CO)	2060	
	Linear Pt_{huc}-(CO)	2036	
	Bridge-bonded CO	1830	
(Pt/Al_2O_3)@In_2O_3	Linear Pt_{wc}-(CO)	2087	3
	Linear Pt_{uc}-(CO)	2066	
Pt_1Sn_1/SiO_2	Linear Pt_{wc}-(CO)	2078	4
	Bridge-bonded CO	1821	
Pt/α-Al_2O_3	Linear Pt_{wc}-(CO)	2098	5
	Linear Pt_{uc}-(CO)	2078	
	Linear Pt_{huc}-(CO)	2035	
PtGa/γ-Al_2O_3	Oxidized Pt	2093	6
	Linear Pt_{iso}-(CO)	2054	

Pt_{wc}: well-coordinated Pt atoms with a metal-metal coordination number of >7.

Pt_{uc}: under-coordinated Pt atoms with a metal-metal coordination number of 6 or 7.

Pt_{huc}: highly under-coordinated Pt atoms with a metal-metal coordination number of <6.

Pt_{iso}: isolated Pt atoms without Pt-Pt bond.

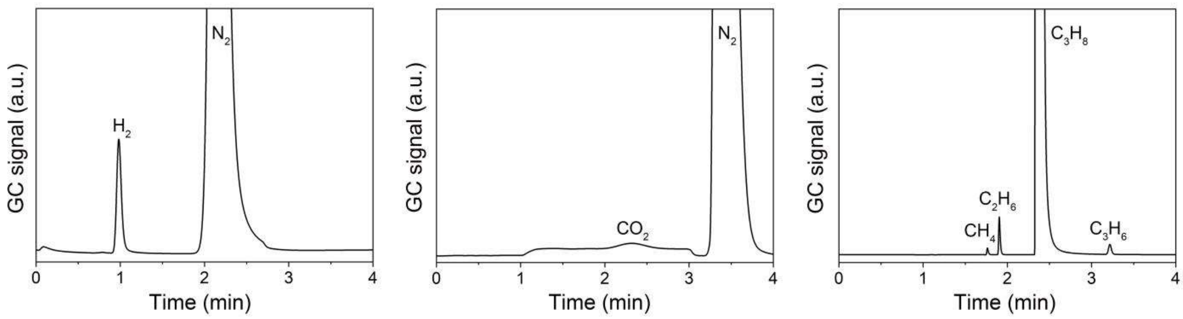

Supplementary Figure 9 | Typical GC spectrum of gas products. Reaction condition: 25 mg of PtSn/γ-Al_2O_3, 5 mL of water, 6 bar (C_3H_8:N_2 = 5:1), 350 ºC, 2 h. Source data are provided as a Source Data file.

Supplementary Table 5 | Molar percentage of liquid and gas products obtained after a standard operation. Typically, 25 mg of PtSn/γ-Al_2O_3 and 5 mL of water were loaded in a 15-mL slurry reactor to operate under 6 bar (C_3H_8:N_2 = 5:1) at 350 ºC for 2 h.

	Gaseous products	Liquid products
Molar production (μmol)	75.2	21.6
Molar percentage (mol%)	77.7	22.3

Supplementary Table 6 | Molar percentage of each product contained in the gas product obtained after a standard operation. Typically, 25 mg of PtSn/γ-Al_2O_3 and 5 mL of water were loaded in a 15-mL slurry reactor to operate under 6 bar (C_3H_8:N_2 = 5:1) at 350 °C for 2 h.

Gaseous products	CO_2	CH_4	C_2H_6	C_3H_6	H_2
Molar percentage (mol%)	10.2	8.3	14.0	5.1	62.4

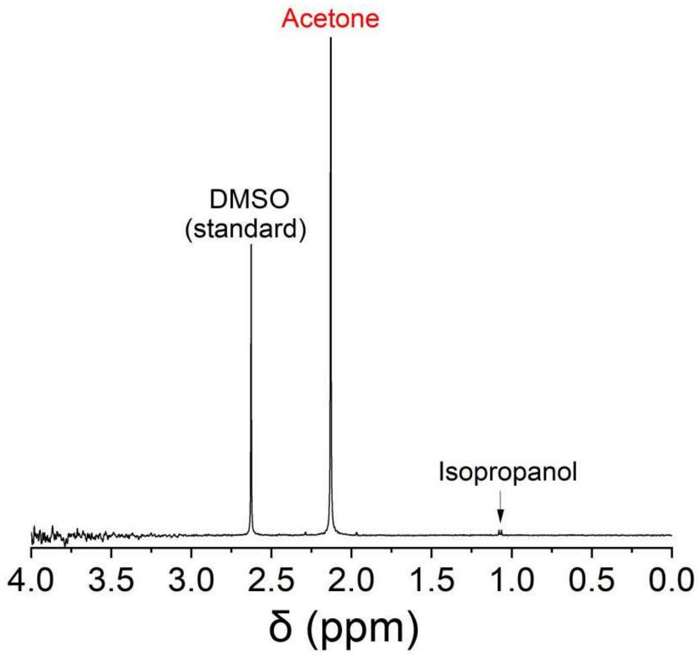

Supplementary Figure 10 | Typical ^{1}H NMR spectrum of liquid products. Reaction condition: 25 mg of PtSn/γ-Al_2O_3, 5 mL of water, 6 bar (C_3H_8:N_2 = 5:1), 350 °C, 2 h. DMSO was used as the standard. Source data are provided as a Source Data file.

Supplementary Table 7 | Molar percentage of each product contained in the liquid product obtained after a standard operation. Typically, 25 mg of PtSn/γ-Al_2O_3 and 5 mL of water were loaded in a 15-mL slurry reactor to operate under 6 bar (C_3H_8:N_2 = 5:1) at 350 ºC for 2 h.

Liquid products	C_3H_6O	C_3H_8O
Molar percentage (mol%)	99.3	0.7

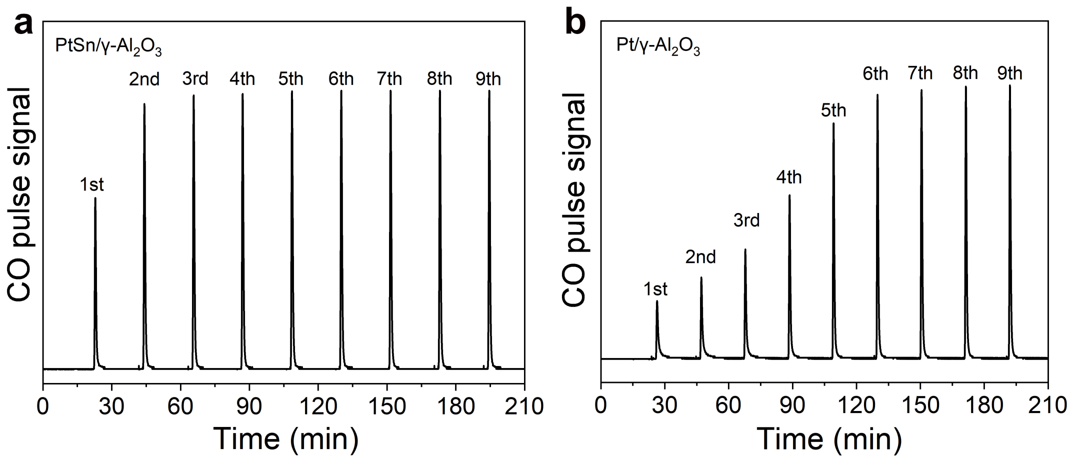

Supplementary Figure 11 | CO pulse titration over PtSn/γ-Al_2O_3 (a) and Pt/γ-Al_2O_3 (b). Source data are provided as a Source Data file.

Supplementary Table 8 | Result of CO pulse titration over PtSn/γ-Al_2O_3 and Pt/γ-Al_2O_3.

CO pulse titration		Unsaturated peak			Saturated peak			
		1st	2nd	3rd	4th	5th	6th	7th
PtSn/γ-Al_2O_3	$A_{Integral}$	17.74	27.64	28.16	28.74	28.81	28.87	28.79
	$A_{Adsorption}$	11.07	1.16	0.64	/	/	/	/
	$n_{Adsorption}$ (μmol)	0.77	0.08	0.05	/	/	/	/
	$\Sigma n_{Adsorption}$ (μmol)		0.90					
Pt/γ-Al_2O_3	$A_{Integral}$	10.97	13.53	15.43	20.51	26.86	29.84	30.29
	$A_{Adsorption}$	19.32	16.75	14.86	9.78	3.43	0.45	/
	$n_{Adsorption}$ (μmol)	1.28	1.11	0.98	0.65	0.23	0.03	/
	$\Sigma n_{Adsorption}$ (μmol)		4.28					

Notes:

As is described in the Methods section, 50 mg of catalysts with a Pt mass loading of 2.71 wt% for PtSn/γ-Al_2O_3 and 2.84 wt% for Pt/γ-Al_2O_3 were used for CO pulse titration. Thus, the moles of total Pt atoms in PtSn/γ-Al_2O_3 catalyst and Pt/γ-Al_2O_3 catalyst were calculated as 6.95 μmol and 7.28 μmol, respectively. The dispersion of Pt was thus calculated as 0.90 μmol ÷ 6.95 μmol × 100% = 12.9% for PtSn/γ-Al_2O_3 catalyst. Meanwhile, the dispersion of Pt in Pt/γ-Al_2O_3 catalyst was thus calculated as 4.28 μmol ÷ 7.28 μmol × 100% = 58.8%.

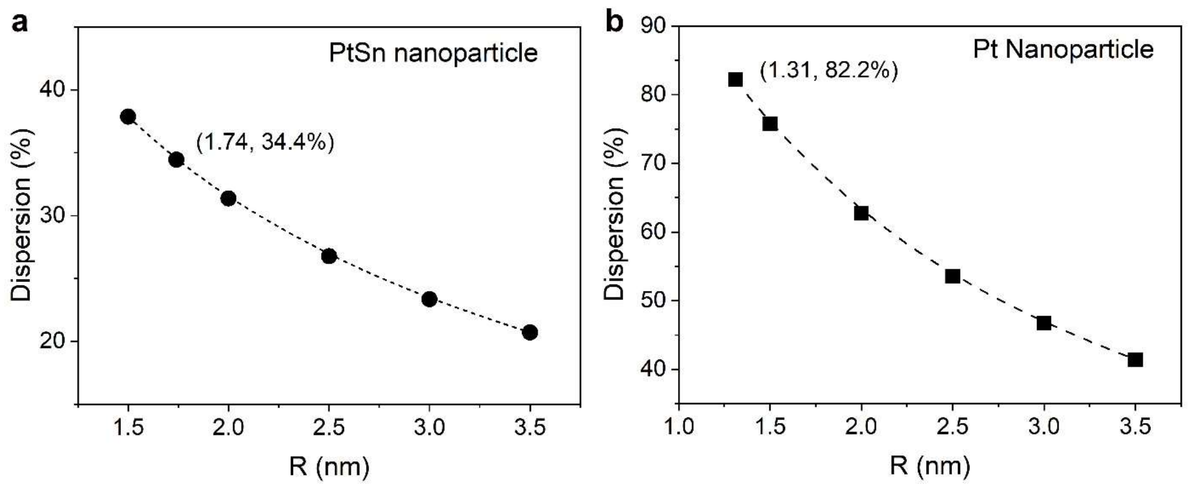

Supplementary Figure 12 | The relationship between the nanoparticle size and Pt dispersion obtained by modeling. (a) PtSn nanoparticle, (b) Pt nanoparticle. Details for the modeling process can be found in Supplementary Note 1. Source data are provided as a Source Data file.

Supplementary Note 1. Discussions on relationship between particle size and Pt dispersion.

We further took an investigation of the relation between nanoparticle size and dispersion to help understand the structural feature of the catalysts. Under the assumption of PtSn as well-defined spherical nanoparticles, we introduced a parameter, P_S, to represent the packing fraction on a surface crystalline plane (the ratio of the plane area occupied by atoms to the total plane area). For the (111) close-packed plane of a face-centered cubic (*fcc*) structure, the area 'covered' by an atom of radius r equals its cross-section (πr^2). The total surface area associated with each atom is $2\sqrt{3}r^2$. Therefore, $P_{S(111),fcc} = \pi r^2/(2\sqrt{3}r^2) = 0.91$. The number of surface atoms, n_s, is calculated as follows:

$$n_s = P_s \frac{4\pi R^2}{\pi r^2} = 4P_s \frac{R^2}{r^2} \quad (1)$$

where R is the radius of the spherical nanoparticle, and r is the atomic radius deduced from the atomic volume ($V_a = 4\pi r^3/3$).

Next, a lattice packing fraction, P_L, is introduced, representing the ratio of the volume of the crystal occupied by atoms to the total volume. Using the example of an *fcc* lattice, the volume of a single atom is $4\pi r^3/3$, and the total volume associated with each atom in the unit cell is $4\sqrt{2}r^3$. Therefore $P_{L,fcc} = (4\pi r^3/3)(4\sqrt{2}r^3) = 0.74$.

To calculate the total number of atoms in the nanoparticle, considering the outer surface composed of many crystalline planes, the volume of the nanoparticle occupied by atoms can be calculated as:

$$\mathrm{n}_i \mathrm{V}_a + \frac{1}{2}\mathrm{n}_s \mathrm{V}_a = \mathrm{P}_L \mathrm{V}_p \quad (2)$$

$$\mathrm{n}_t = \mathrm{n}_i + \mathrm{n}_s \quad (3)$$

where V_a is the atomic volume, and V_p is the volume of the particle. Thus,

$$\mathrm{n}_t = P_L \frac{R^3}{r^3} + 2P_s \frac{R^2}{r^2} \quad (4)$$

Assuming a surface atomic ratio of Pt/Sn as 1:1 and the metals share a same atomic radius of r_{Pt} = 0.138 nm, the dispersion of Pt can be calculated as:

$$dispersion_{PtSn\ nanoparticle} = \frac{1}{2} \times \frac{\mathrm{n}_s}{\mathrm{n}_t} = \frac{2P_s}{P_L \frac{R}{r} + 2P_s} = \frac{1}{1.094 \times R + 1} \quad (5)$$

$$dispersion_{Pt\ nanoparticle} = \frac{\mathrm{n}_s}{\mathrm{n}_t} = \frac{2P_s}{P_L \frac{R}{r} + 2P_s} = \frac{2}{1.094 \times R + 1} \quad (6)$$

To this end, a clear relationship between dispersion and nanoparticle size can be illustrated in Supplementary Figure 12.

As illustrated, the increase of the nanoparticle size induces the significant decrease of the Pt dispersion. With an average nanoparticle size (R) of 1.74 nm for PtSn nanoparticles and 1.31 nm for Pt nanoparticles (Supplementary Fig. 2), we can deduce a theoretical Pt dispersion of 34.4%

for PtSn nanoparticles and 82.2% for Pt nanoparticles, based on equation 5 and 6, respectively. Of note, these values were apparently larger than the value (12.9% and 58.8%) based on CO pulse titration. The difference can be attributed to the imperfectly spherical morphology of the nanoparticles, and more importantly, the close intact of the nanoparticles to the γ-Al_2O_3 support, which hinders part of the surface atoms from adsorbing CO molecules. We also exclude the possibility of the deviation of Pt/Sn ratio to 1:1 that caused the decrease, since the existence of isolated SnO_x nanoparticles indicated a higher Pt/Sn ratio in the surface which leads to a higher Pt dispersion. For comparison, previously reported Pt/Al_2O_3 catalyst synthesized *via* traditional incipient-wetness impregnation method exhibited a Pt dispersion of 70~85% with Pt nanoparticle size of 1.2~1.4 nm, which is very close to the calculated 82.2% value based on the above model [7,8]. Therefore, we ascribed the relatively low dispersion value mainly to the close intact between nanoparticles and support, which provides abundant interfaces for successive catalytic processes and ensures a good stability.

Supplementary Table 9 | Comparison of propane conversion. Comparison between the propane conversion during wet reforming over PtSn/γ-Al_2O_3 and the kinetic analysis. Details for the calculation process can be found in Supplementary Note 2.

Reaction conditions	Propane conversion	Moles of converted propane molecules	Apparent reaction rate constant (*k*)	Apparent reaction rate (*R*)
5 bar of C_3H_8 and 1 bar of N_2, 350 °C	1.84% (2 h)	37.13 μmol	0.007 $bar^{-0.12}$ h^{-1}	0.141 bar h^{-1}
3 bar of C_3H_8 and 1 bar of N_2, 350 °C	2.69% (2 h)	32.57 μmol	0.011 $bar^{-0.12}$ h^{-1}	0.126 bar h^{-1}
0.2 bar of C_3H_8 and 0.8 bar of N_2, 350 °C	25.60% (2 h)	20.67 μmol	0.161 $bar^{-0.12}$ h^{-1}	0.087 bar h^{-1}

Supplementary Note 2. Calculation of forward reaction rate.

We have also calculated the forward reaction rate based on the kinetic equations of the propane-to-acetone process. Figure 3c indicates that the apparent reaction order for propane steam reforming to produce acetone is 1.12. From the apparent reaction order, the reaction kinetic rate equation can be written as:

$$R = -\frac{dp_{C_3H_8}}{dt} = k \cdot {p_{C_3H_8}}^{1.12} \tag{7}$$

$$-\frac{dp_{C_3H_8}}{{p_{C_3H_8}}^{1.12}} = k\ \mathrm{dt} \tag{8}$$

$$\frac{1}{0.12\ {p_{C_3H_8}}^{0.12}} = kt + M \tag{9}$$

where R is the apparent reaction rate, p_{C3H8} is the partial pressure of propane at the certain time, t is the reaction time, k is the apparent reaction rate constant, and M is the integral constant.

At t = 0, the initial pressures under three reaction conditions are 14.6 bar, 8.8 bar, and 0.58 bar, respectively, based on the Charles' law, $P = P_0\,(1 + \mathrm{T}/273)$. By substituting these initial values into equation 9, the integral constant terms M under these three conditions are found to be 6.04, 6.42, and 8.89.

At t = 2 h, the final state corresponding to the conversion rate of C_3H_8 is calculated for the respective conditions. These values, along with the integral constant terms M mentioned above, are substituted into equation 9 to determine the corresponding apparent reaction rate constant, k, and apparent reaction rate, R, as shown in Supplementary Table 9.

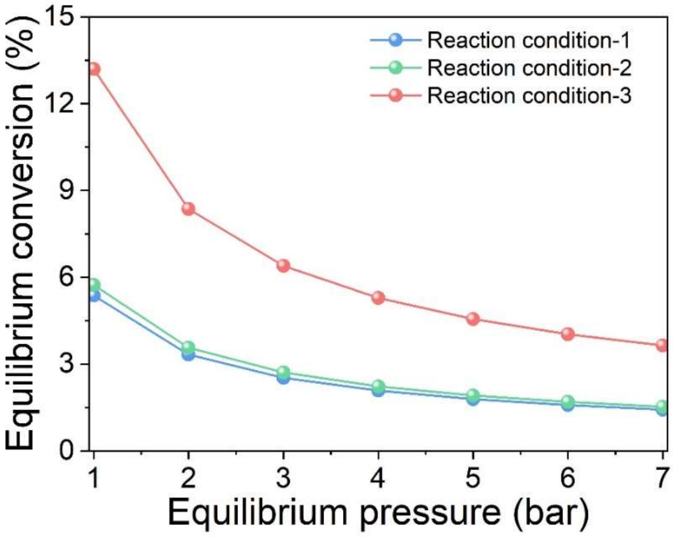

Supplementary Figure 13 | Dependence of equilibrium conversion on equilibrium pressure for the propane-wet-reforming process. Details for the calculation process can be found in Supplementary Note 3. Source data are provided as a Source Data file.

Supplementary Note 3. Calculation of equilibrium conversion and energies.

The calculations of equilibrium conversion and Gibbs energy were carried out by using HSC Chemistry 6 software.

Firstly, we calculated the standard reaction Gibbs energy ($\Delta_r G^\Theta$) for the propane-wet-reforming process at 350 °C. Under ideal condition at 350 °C, the saturated vapor pressure of water is 16521 kPa, while the density of liquid water is 0.59 g mL^{-1} under 16521 kPa at 350 °C. The volume of added water at room temperature is 5 mL, while that of the reactor is 15 mL. It was estimated that the water in the slurry reactor existed in the form of liquid at 350 °C, containing 0.47 g of gaseous water and 4.54 g of liquid water. Considering that the catalyst was immersed in the water, we assumed the water at the liquid state during the calculation. Considering that acetone can be completely dissolved in the water and that the concentration of acetone was extremely low during the experiments, we assumed the acetone as the solute in water. Taking the above assumptions into account, we input the reaction equation of "C_3H_8 (g) + H_2O (l) → C_3H_6O (aq) + $2H_2$ (g)" in the module of HSC Chemistry 6-Reaction Equation. Thus, the value of $\Delta_r G^\Theta$ at 350 °C was calculated as 44.74 kJ mol^{-1}, while the corresponding standard equilibrium constant is 1.776 × 10^{-4}.

Then, we calculated the equilibrium conversion of propane under different conditions by using the module of HSC Chemistry 6-Equilibrium Compositions. C_3H_8(PPEg), H_2O, C_3H_6O(PREg), and H_2(g) were selected as the reactants. The reaction temperature was set at 350 °C. The initial compositions of reactants were listed in as follows and input into the calculation module. The equilibrium conversion of propane was obtained and shown in Supplementary Figure 13. As shown in Supplementary Figure 13, the equilibrium conversion of propane decreases with the increase of the propane partial pressure. As such, the equilibrium conversion of propane in condition 3 is much higher than those in conditions 1 and 2, corresponding to the experimental value. It is worth noting that the equilibrium conversion of propane varies slightly under the propane partial pressure above 5 bar for conditions 1 and 2 because the propane conversion is rather low. This phenomenon is consistent with the experimental results where the conversion of propane did not increase with increasing the partial pressure of propane in the reaction system (Supplementary Table 9).

Condition	C_3H_8	H_2O	N_2	C_3H_6O	H_2
1	2.05 mmol	0.28 mol	0.41 mmol	0 mmol	0 mmol
2	1.23 mmol	0.28 mol	0.41 mmol	0 mmol	0 mmol
3	0.08 mmol	0.28 mol	0.33 mmol	0 mmol	0 mmol

Since these three conditions are at the same reaction temperature, they have the same standard equilibrium constant (equation 10). The corresponding variation of the compositions for the reaction C_3H_8 (g) + H_2O (l) → C_3H_6O (aq) + $2H_2$ (g) are listed in as follows.

$$K^\theta = \frac{([H_2]/p^\theta)^2[C_3H_6O]/c^\theta}{[C_3H_8]/p^\theta} \quad (10)$$

	C_3H_8 (g)/(bar)	C_3H_6O (aq)/(mol L^{-1})	H_2 (g)/(bar)	N_2 (g)/(bar)
Initial	p_I	0	0	pN
Change	x	$0.0183x$ [a]	$2x$	pN
Eq.	$p_I - x$	$0.0183x$	$2x$	pN

[a]Calculation based on the equation 11.

$$c = \frac{n}{V_l} = \frac{pV_g}{RTV_l} = \frac{100x \times 0.0073}{8.314 \times 623 \times 0.0077} \text{ mol L}^{-1} = 0.0183x \text{ mol L}^{-1} \quad (11)$$

At 350 °C, the volume of water is 7.7 mL, while the volume of total gas is 7.3 mL. Based on the equation PV/T = constant, the initial partial pressures of propane under conditions 1-3 are 14.6, 8.8, and 0.58 bar, respectively, while those of N_2 are 2.92, 2.92, and 2.33 bar, respectively. We plug these values into equation 1 and obtain equation 3. After solving the equation 12, the values of x under conditions 1-3 are 0.15, 0.13, and 0.05, respectively. The corresponding conversions are 1.03%, 1.48%, and 8.62%, respectively.

$$1.776 \times 10^{-4} = \frac{0.0183x \times (2x)^2}{p_I - x} \quad (12)$$

Finally, we calculated non-standard reaction Gibbs energy ($\Delta_r G$) based on equations 13 and 14.

$$\Delta_r G = \Delta_r G^\Theta + nRT\, ln(p/p^\Theta) \quad \text{(Gas)} \quad (13)$$

$$\Delta_r G = \Delta_r G^\Theta + nRT\, ln(c/c^\Theta) \quad \text{(Solution)} \quad (14)$$

Since the initial partial pressure of H_2 and the initial concentration of acetone are zero, it is meaningless to calculate $\Delta_r G$. As such, we calculated the values of $\Delta_r G$ after 2-h reaction based on equation 15 to judge whether the reaction reaches the equilibrium state. In equation 15, Q is the reaction quotient defined in equation 16.

$$\Delta_r G = \Delta_r G^\Theta + nRT\, lnQ \quad (15)$$

$$Q = \frac{(p(H_2)/p^\theta)^2\, c(C_3H_6O)/c^\theta}{p(C_3H_8)/p^\theta} \quad (16)$$

We plug the compositions after 2-h reaction and the standard reaction Gibbs energy into equation 16. The corresponding values of $\Delta_r G$ are calculated as -10.70 kJ/mol, -5.76 kJ/mol, and +2.66 kJ mol^{-1}.

Supplementary Table 10 | Comparison between the propane conversion and the thermodynamic equilibrium conversion of propane during wet reforming. Equilibrium calculations were performed through HSC Chemistry 6 software, which utilizes a Gibbs free energy minimization algorithm. Reaction condition: 5 mL of H_2O, 350 °C, 2 h.

Condition	Gas composition	Propane conversion	Acetone selectivity	Yield of acetone	Thermodynamic equilibrium conversion	Gibbs free energy change
1	5 bar of C_3H_8, 1 bar of N_2	1.84%	57.82%	1.06%	1.03%	-10.70 kJ/mol
2	3 bar of C_3H_8, 1 bar of N_2	2.69%	37.53%	1.01%	1.48%	-5.76 kJ/mol
3	0.2 bar of C_3H_8, 0.8 bar of N_2	25.60%	35.85%	9.18%	8.62%	+2.66 kJ/mol

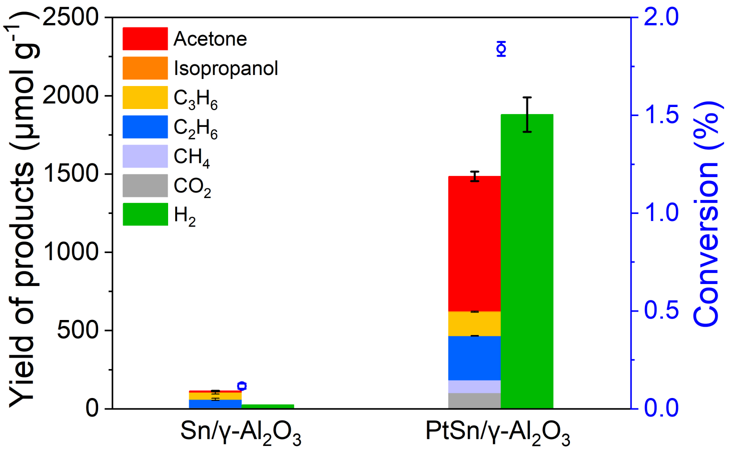

Supplementary Figure 14 | Yield of products for propane-wet-reforming over Sn/γ-Al_2O_3 and PtSn/γ-Al_2O_3. Typically, 25 mg of the catalyst was operated in 5 mL of water under 6 bar (C_3H_8:N_2 = 5:1) at 350 °C for 2 h. Error bars represent the standard deviation from three independent measurements. Source data are provided as a Source Data file.

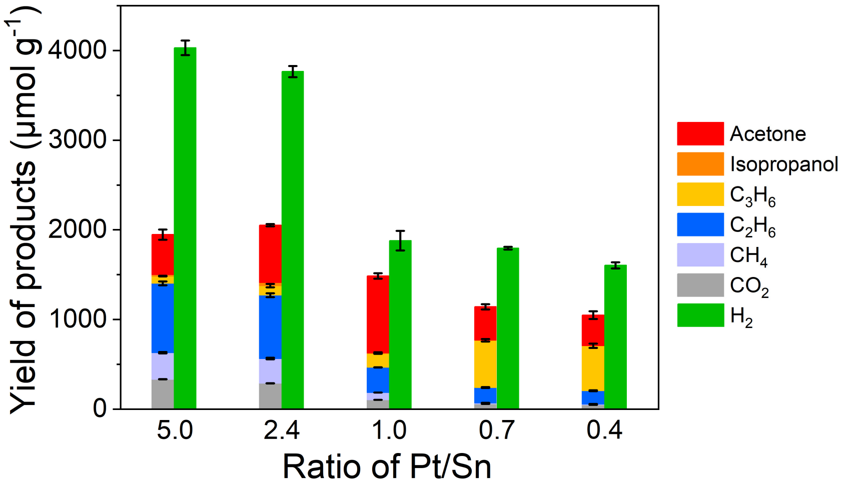

Supplementary Figure 15 | Dependence of catalytic properties of PtSn/γ-Al_2O_3 catalysts on Pt/Sn ratios. Typically, 25 mg of samples with different ratio of Pt/Sn and 5 ml of water were loaded in a 15-mL slurry reactor with the stirring speed of 600 rpm to operate under 6 bar (C_3H_8:N_2 = 5:1) at 350 °C for 2 h. Error bars represent the standard deviation from three independent measurements. Source data are provided as a Source Data file.

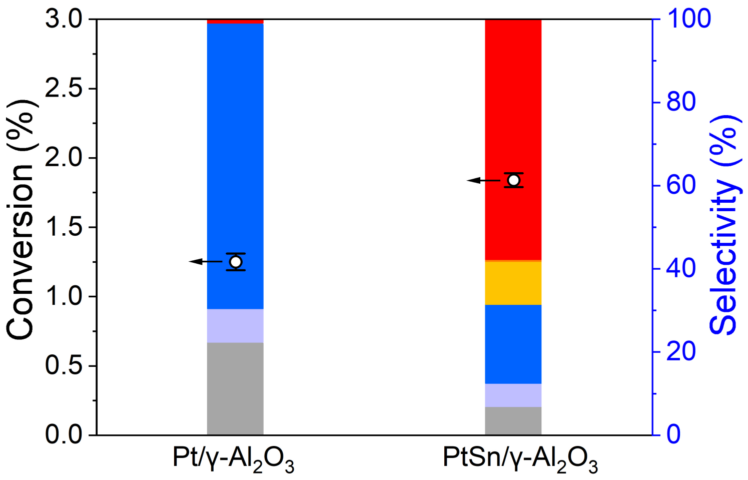

Supplementary Figure 16 | Comparison of product distribution at similar conversions over Pt/γ-Al_2O_3 and PtSn/γ-Al_2O_3. For Pt/γ-Al_2O_3, 25 mg of Pt/γ-Al_2O_3 and 5 mL of water were loaded in a 15-mL slurry reactor to operate under 6 bar (C_3H_8:N_2 = 5:1) at 350 °C for 10 min. For PtSn/γ-Al_2O_3, 25 mg of PtSn/γ-Al_2O_3 and 5 mL of water were loaded in a 15-mL slurry reactor to operate under 6 bar (C_3H_8:N_2 = 5:1) at 350 °C for 2 h. Error bars represent the standard deviation from three independent measurements. Source data are provided as a Source Data file.

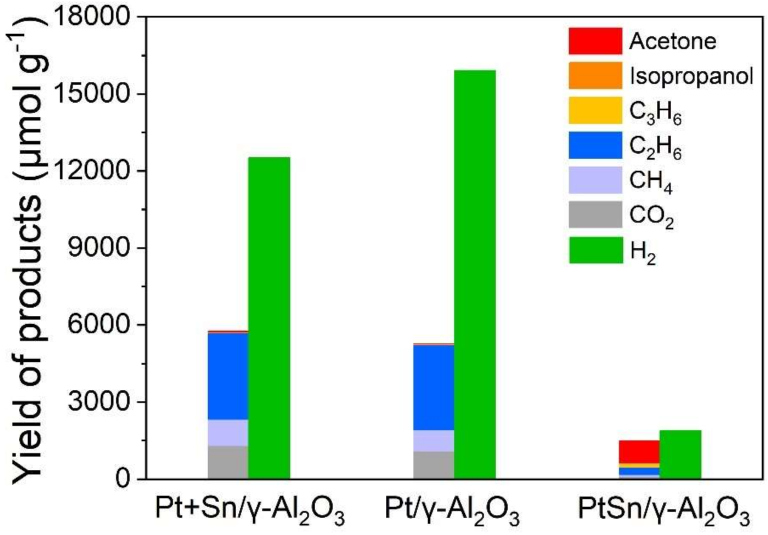

Supplementary Figure 17 | Comparison in catalytic properties of Pt+Sn/γ-Al_2O_3, Pt/γ-Al_2O_3, and PtSn/γ-Al_2O_3. Typically, 25 mg of sample and 5 ml of water were loaded in a 15-mL slurry reactor with the stirring speed of 600 rpm to operate under 6 bar (C_3H_8:N_2 = 5:1) at 350 ºC for 2 h. Source data are provided as a Source Data file.

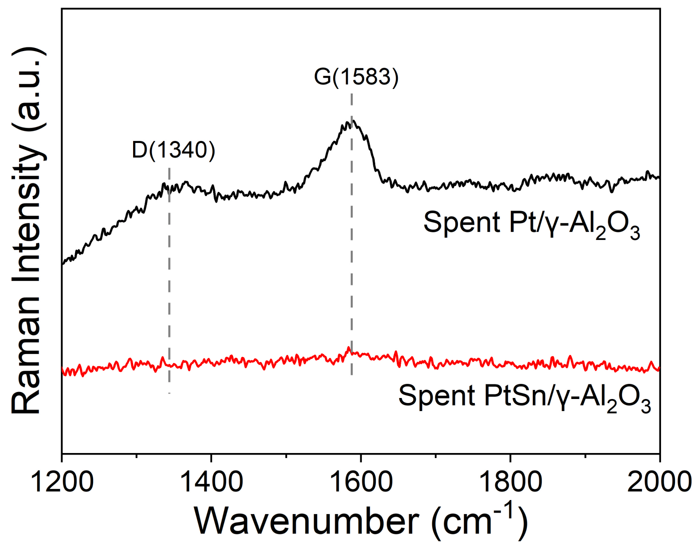

Supplementary Figure 18 | Raman spectra of spent PtSn/γ-Al_2O_3 and spent Pt/γ-Al_2O_3. Source data are provided as a Source Data file.

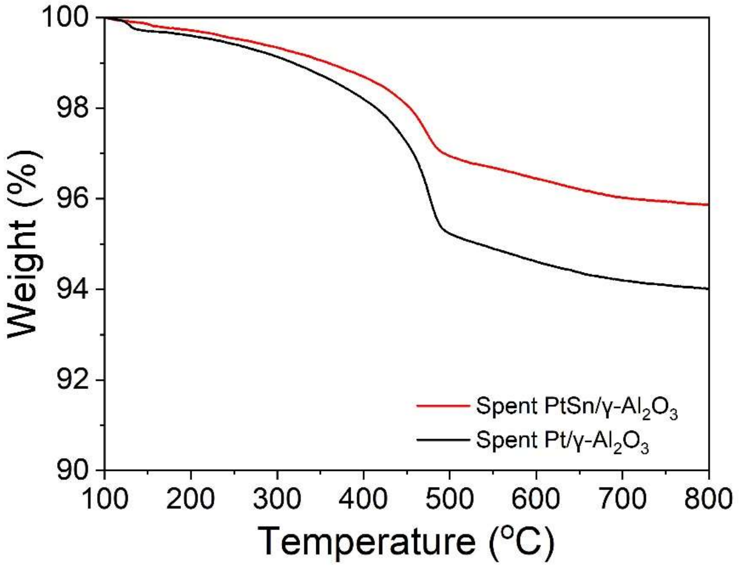

Supplementary Figure 19 | TG profiles of spent PtSn/γ-Al_2O_3 and spent Pt/γ-Al_2O_3. Source data are provided as a Source Data file.

Supplementary Table 11 | Mass balance of carbon species for PtSn/γ-Al_2O_3 and Pt/γ-Al_2O_3.

Sample	Total Consumed propane derived from GC analysis before and after reaction	Consumed propane derived from produced molecules via GC, ^{1}H NMR analysis	Consumed propane derived from produced Coke via TG analysis	Modified carbon balance
PtSn/γ-Al_2O_3	99.03 μmol	37.12 μmol	26.54 μmol	64.3%
Pt/γ-Al_2O_3	256.86 μmol	131.80 μmol	38.40 μmol	66.3%

Supplementary Note 4. Discussions on the mass balance.

We carried out TG analysis of the spent catalysts. The weight losses of spent PtSn/γ-Al_2O_3 and spent Pt/γ-Al_2O_3 were 4.14% and 5.99%, respectively (Supplementary Figure 19). Assuming that the coke exists in the form of C_6H_6 (M_w = 78), we can calculate the amount of consumed propane for coke formation as:

25 mg × 4.14% ÷ 78 g/mol × 2 = 26.5 μmol (For PtSn/γ-Al_2O_3)
25 mg × 5.99% ÷ 78 g/mol × 2 = 38.4 μmol (For Pt/γ-Al_2O_3)

Then, we calculated the carbon balance based on a modified equation (eq. 17) for reactions with low conversions[9].

$$\text{Modified carbon balance} = \frac{\sum n_{produced}}{\sum n_{consumed}} \times 100\% \qquad (17)$$

The results were presented in Supplementary Table 11. Of note, the obtained carbon balance for PtSn/γ-Al_2O_3 and Pt/γ-Al_2O_3 are 64.3% and 66.3%, respectively, which are apparently smaller than the ideal 100% value. Since the quantification methodology including GC and ^{1}H NMR analysis in this study strictly calibrated by standard curves. We suspect that the main reason for the underestimated carbon balance to the exfoliation of coke species under the harsh hydrothermal conditions. As previously reported, aliphatic coke species are preferred to be formed in PtSn/Al_2O_3 while polyaromatics are preferred to be formed in Pt/Al_2O_3[10]. The dilution of the adjacent Pt atoms by Sn incorporation provides even less anchoring site for aliphatic coke species, enabling easier detachment process from the metal surface by hydration or hydrogenation. These results are consistent with the observed fewer coke amount in spent PtSn/γ-Al_2O_3 than Pt/γ-Al_2O_3. It also rationalized that the preferential formation of aliphatic coke species results in the negligible D and G band signals in the Raman spectrum of PtSn/γ-Al_2O_3, while considerable coke species are quantified through TG analysis. Unfortunately, these detached carbon fragments are too low in productivity to be collected for analysis.

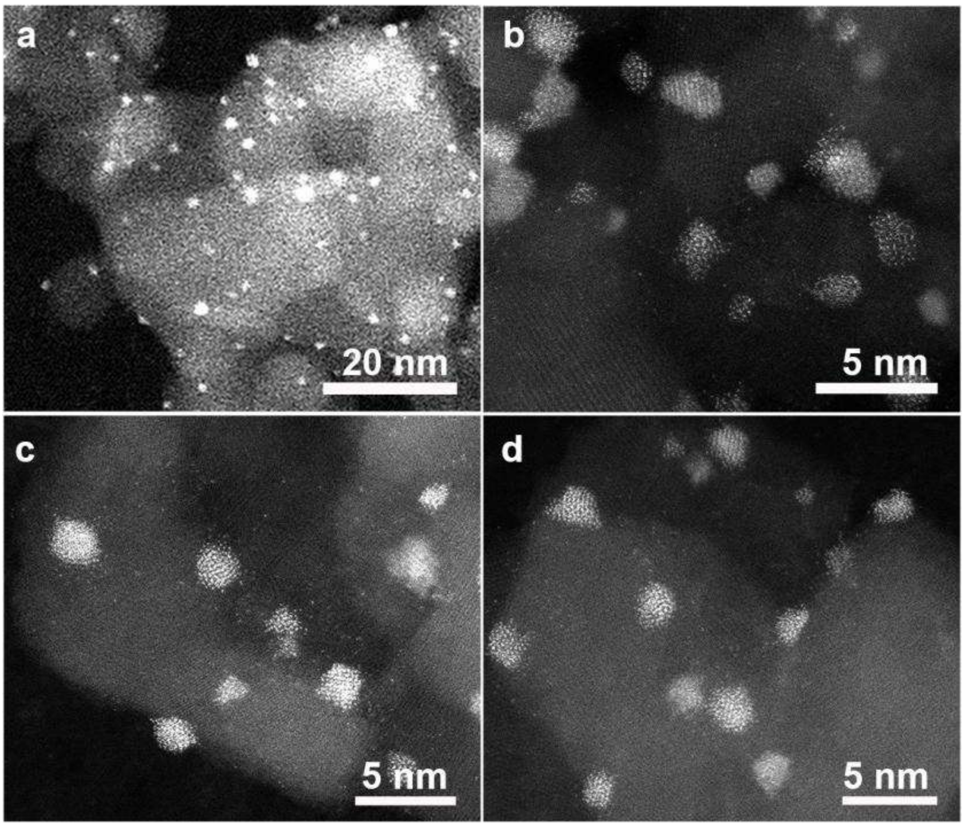

Supplementary Figure 20 | Magnified HAADF-STEM image of spent PtSn/γ-Al_2O_3. (a) HAADF-STEM image of spent PtSn/γ-Al_2O_3 at low magnification. (b-d) HAADF-STEM images of spent PtSn/γ-Al_2O_3 at high magnification.

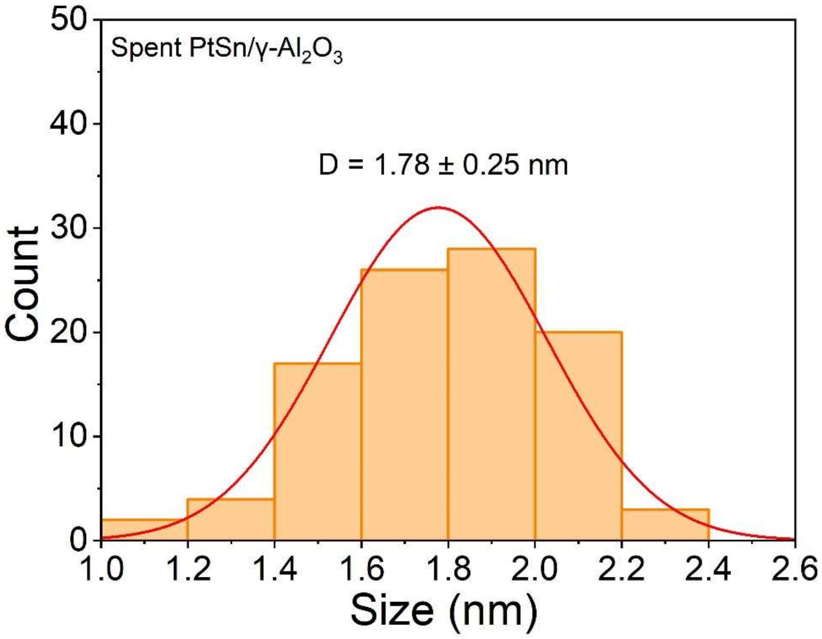

Supplementary Figure 21 | Size distribution of metal nanoparticles in spent PtSn/γ-Al_2O_3. Source data are provided as a Source Data file.

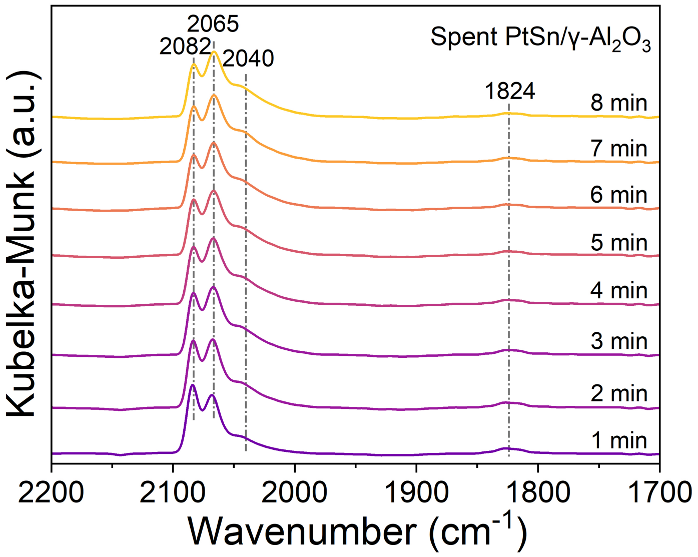

Supplementary Figure 22 | CO-DRIFTS spectra of spent PtSn/γ-Al_2O_3. Source data are provided as a Source Data file.

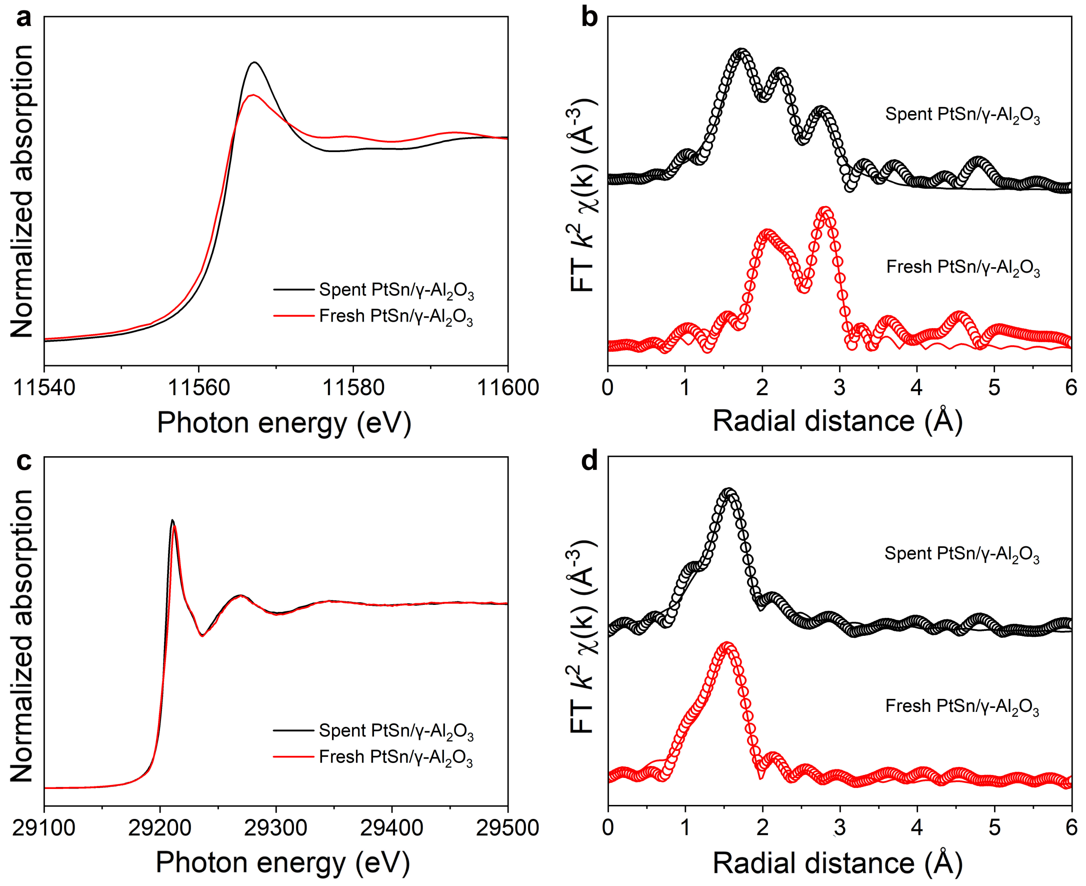

Supplementary Figure 23 | XAS analysis of spent PtSn/γ-Al_2O_3. (a) Pt L_3-edge XANES spectra and (b) the corresponding Pt L_3-edge EXAFS spectra of fresh and spent PtSn/γ-Al_2O_3. (c) Sn K-edge XANES spectra and (d) the corresponding Sn K-edge EXAFS spectra of fresh and spent PtSn/γ-Al_2O_3. Source data are provided as a Source Data file.

Supplementary Table 12 | EXAFS fitting parameters at the Pt L_3-edge for spent PtSn/γ-Al_2O_3.

Sample	Shell	N[a]	R(Å)[b]	σ^2(Å^2)[c]	ΔE_0 (eV)[d]	R factor
Fresh PtSn/γ-Al_2O_3	Pt-O	0.3±0.1	1.95±0.01	0.0155±0.0052	3.9±2.0	0.0020
	Pt-Sn	1.6±0.2	2.70±0.02	0.0064±0.0030		
	Pt-Pt	4.3±0.4	2.74±0.01	0.0013±0.0012		
Spent PtSn/γ-Al_2O_3	Pt-O	0.8±0.1	2.00±0.01	0.0030±0.0018	8.6±1.5	0.0023
	Pt-Sn	1.6±0.5	2.71±0.03	0.0064[e]		
	Pt-Pt	3.8±0.8	2.77±0.01	0.0072±0.0018		

[a] *N*: coordination numbers.
[b] *R*: bond distance.
[c] σ^2: Debye-Waller factors.
[d] ΔE_0: the inner potential correction.
[e] The σ^2 value of the Pt-Sn path was fixed at 0.0064 for the fitting due to the following reason. We observed a marginal decline in the signal-to-noise ratio in the spectral data of the spent sample. To ensure the quality of fitting, we set the σ^2 of the Pt-Sn path to 0.0064, a value derived from reference to static fitting results. The rationale behind fixing the Pt-Sn path arose from our analysis of operando data, wherein we noticed a more pronounced peak intensity variation in the first shell of the sample's R space compared to the static condition. This variation was mainly attributed to Pt-O interaction. Furthermore, the coordination number of Pt-Pt has the highest overall proportion. As a result, rather than imposing constraints on Pt-O and Pt-Pt, we fixed the σ^2 specifically for the Pt-Sn path.

Supplementary Table 13 | EXAFS fitting parameters at the Sn K-edge for spent PtSn/γ-Al_2O_3.

Sample	Shell	N[a]	R(Å)[b]	σ^2(Å^2)[c]	ΔE_0(eV)[d]	R factor
Fresh PtSn/γ-Al_2O_3	Sn-O	5.0±0.5	2.01±0.02	0.0045±0.0035	-0.3±0.4	0.0048
Spent PtSn/γ-Al_2O_3	Sn-O	5.1±0.5	2.02±0.01	0.0054±0.0016	2.2±1.5	0.0049

[a] *N*: coordination numbers.

[b] *R*: bond distance.

[c] σ^2: Debye-Waller factors.

[d] ΔE_0: the inner potential correction.

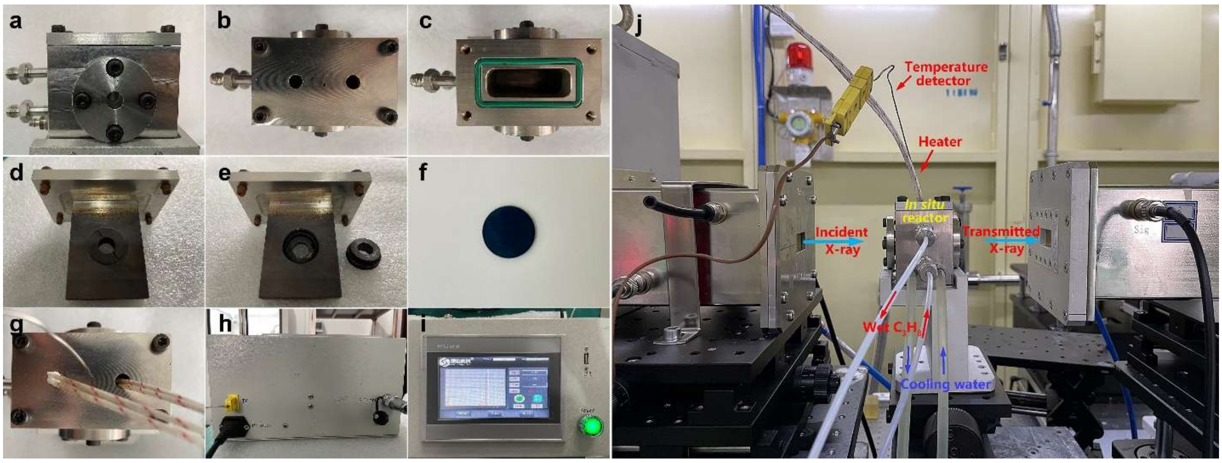

Supplementary Figure 24 | Photograph of the setup for the *operando* XAS. (a) Front view and (b) top view of cell. (c) Top view of chamber of cell. (d) Locked and (e) unlocked sample stage. (f) Sample compressed into a disc with 10 mm in diameter under 2 MPa for 2 min. (g) Top view of cell connected with the heater and K-type thermocouple. (h) Back view and (i) front view of console application for external heating and measurement of the sample temperature. (j) Front view of installed setup for the *operando* XAS test, consists of reaction cell, gas channels for wet propane, cooling water channel, Be windows and heater control system.

Supplementary Table 14 | EXAFS fitting parameters at the Pt L_3-edge for *operando* PtSn/γ-Al_2O_3.

Sample	Shell	N^a	R (Å)[b]	σ^2(Å^2)[c]	ΔE_0 (eV)[d]	R factor
Fresh sample	Pt-O	0.3±0.1	1.95±0.01	0.0155±0.0052	3.9±2.0	0.0020
	Pt-Sn	1.6±0.2	2.70±0.02	0.0064±0.0030		
	Pt-Pt	4.3±0.4	2.74±0.01	0.0013±0.0012		
Operando sample	Pt-O	0.5±0.7	2.02±0.07	0.0019±0.0011	6.2±0.8	0.0048
	Pt-Sn	1.3±0.7	2.68±0.11	0.0064[e]		
	Pt-Pt	3.8±1.4	2.73±0.01	0.0059±0.0082		

[a] N: coordination numbers.

[b] R: bond distance.

[c] σ^2: Debye-Waller factors.

[d] ΔE_0: the inner potential correction.

[e] The σ^2 value of the Pt-Sn path was fixed at 0.0064 for the fitting due to the following reason. We observed a marginal decline in the signal-to-noise ratio in the spectral data of operando samples. To ensure the quality of fitting, we set the σ^2 of the Pt-Sn path to 0.0064, a value derived from reference to static fitting results. The rationale behind fixing the Pt-Sn path arose from our analysis of operando data, wherein we noticed a more pronounced peak intensity variation in the first shell of the sample's R space compared to the static condition. This variation was mainly attributed to Pt-O interaction. Furthermore, the coordination number of Pt-Pt has the highest overall proportion. As a result, rather than imposing constraints on Pt-O and Pt-Pt, we fixed the σ^2 specifically for the Pt-Sn path.

Supplementary Table 15 | EXAFS fitting parameters at the Sn K-edge for *operando* PtSn/γ-Al_2O_3.

Sample	Shell	N^a	R (Å)^b	σ^2(Å^2)^c	ΔE_0(eV)^d	R factor
Fresh sample	Sn-O	5.0±0.5	2.01±0.02	0.0045±0.0035	-0.3±0.4	0.0048
Operando sample	Sn-O	5.1±0.6	2.02±0.01	0.0065±0.0015	3.4±1.6	0.0060

[a] N: coordination numbers.
[b] R: bond distance.
[c] σ^2: Debye-Waller factors.
[d] ΔE_0: the inner potential correction.

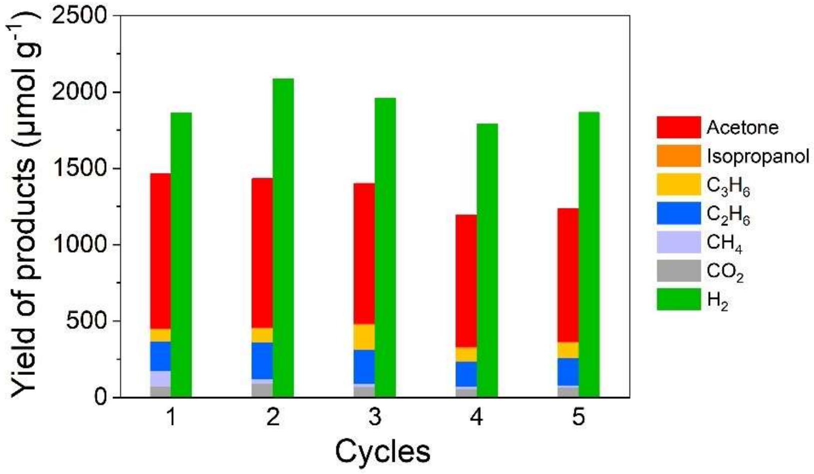

Supplementary Figure 25 | Stability tests over PtSn/γ-Al_2O_3 for different cycles. Typically, 25 mg of PtSn/γ-Al_2O_3 (>60 mesh) and 5 ml of water were loaded in a 15-mL slurry reactor with the stirring speed of 600 rpm to operate under 6 bar (C_3H_8:N_2 = 5:1) at 350 °C for 2 h. Source data are provided as a Source Data file.

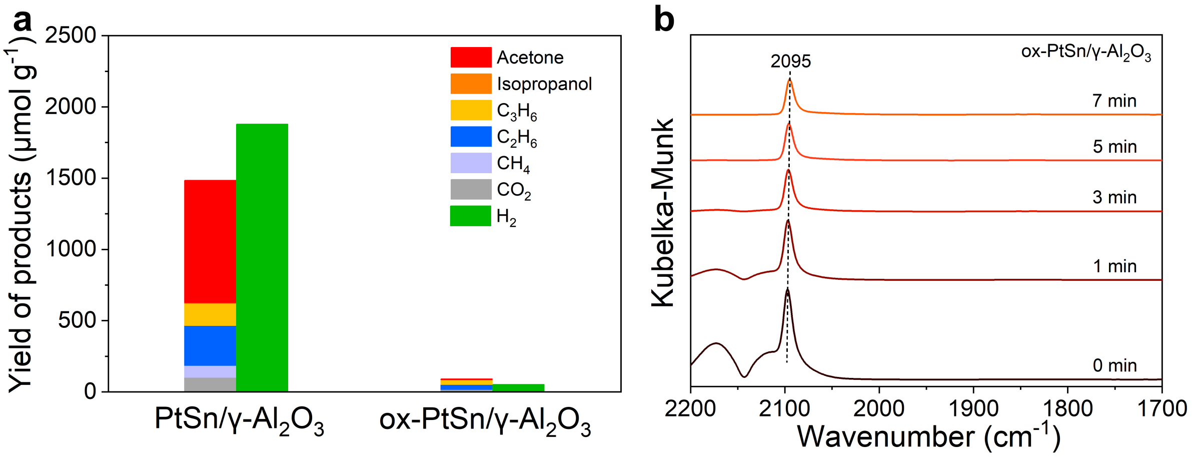

Supplementary Figure 26 | (a) Comparison of the catalytic performance between PtSn/γ-Al_2O_3 and deliberately oxidized PtSn/γ-Al_2O_3 (denoted as ox-PtSn/γ-Al_2O_3). (b) CO-DRIFTS spectra of ox-PtSn/γ-Al_2O_3. The PtSn/γ-Al_2O_3 was calcined in air for 2 h at 750 °C to oxidize surface metallic Pt species (denoted as ox-PtSn/γ-Al_2O_3). Source data are provided as a Source Data file.

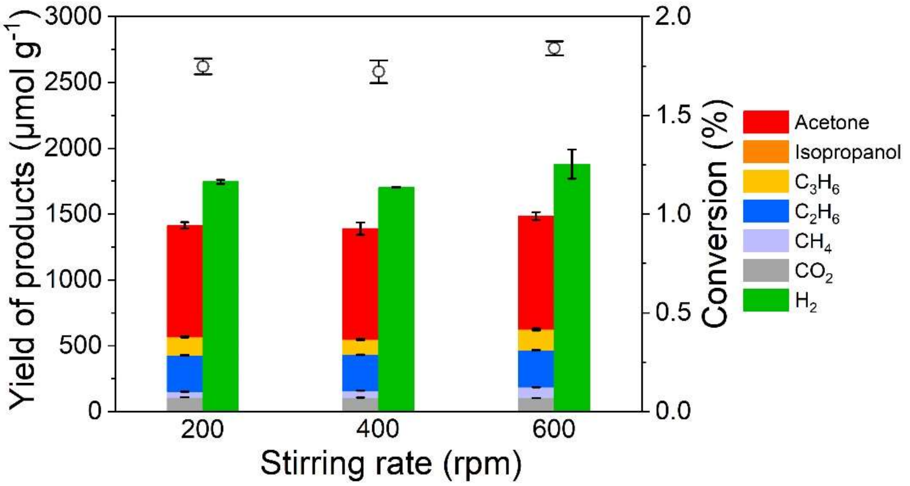

Supplementary Figure 27 | Dependence of products and conversion on the stirring rate. Typically, 25 mg of PtSn/γ-Al_2O_3 (>60 mesh) and 5 mL of water were loaded in a 15-mL slurry reactor with different stirring speeds to operate under 6 bar (C_3H_8:N_2 = 5:1) at 350 °C for 2 h. Error bars represent the standard deviation from three independent measurements. Source data are provided as a Source Data file.

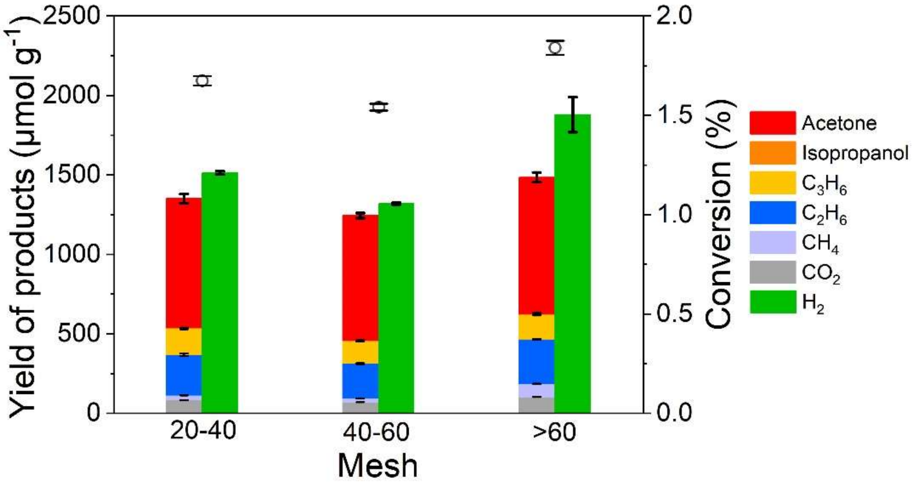

Supplementary Figure 28 | Dependence of products and conversion on the mesh. Typically, 25 mg of PtSn/γ-Al_2O_3 in different meshes and 5 mL of water were loaded in a 15-mL slurry reactor with the stirring speed of 600 rpm to operate under 6 bar (C_3H_8:N_2 = 5:1) at 350 ºC for 2 h. Error bars represent the standard deviation from three independent measurements. Source data are provided as a Source Data file.

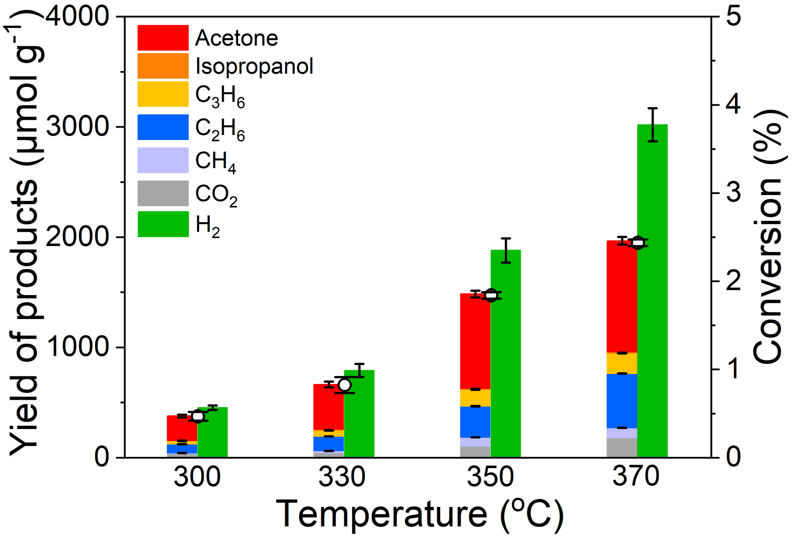

Supplementary Figure 29 | Catalytic properties of PtSn/γ-Al_2O_3 during wet reforming at different temperatures for 2 h. Error bars represent the standard deviation from three independent measurements. Source data are provided as a Source Data file.

Supplementary Table 16. Typical reactions for CO_2 production and C-C cracking.

Reaction		$\Delta H^{\ominus}$ (kJ/mol)
$C_3H_6O + 5H_2O \rightarrow 3CO_2 + 8H_2$	steam reforming of acetone	+244
$C_3H_6 + 6H_2O \rightarrow 3CO_2 + 9H_2$	steam reforming of propene	+251
$C_3H_8 + 6H_2O \rightarrow 3CO_2 + 10H_2$	steam reforming of propane	+375
$C_2H_6 + 6H_2O \rightarrow 3CO_2 + 9H_2$	steam reforming of ethane	+251
$C_3H_8 \rightarrow C_2H_6 + H_2 + C$ (coke)	cracking	+19
$C_2H_6 \rightarrow CH_4 + H_2 + C$ (coke)	cracking	+9

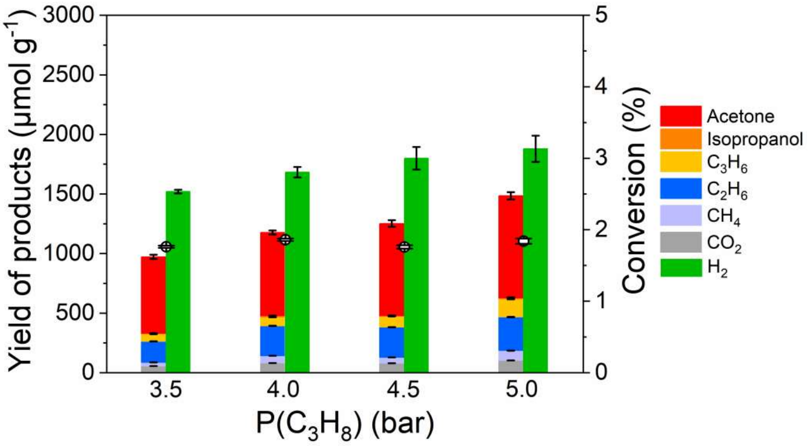

Supplementary Figure 30 | Yields of products over PtSn/γ-Al_2O_3 at different partial pressure of propane at 350 °C for 2 h. Error bars represent the standard deviation from three independent measurements. Source data are provided as a Source Data file.

Supplementary Note 5. Discussion on reaction kinetics.

The reaction order with respect to propane was 1.12. Thus, we can approximately regard it as the first-order reaction with respect to propane. The steady conversion regardless of the partial pressure of propane was attributed to the first-order reaction with respect to propane and the low conversion (~2%) of propane. The differential form of the first-order reaction is written as equation 18 and rewritten as equation 19.

$$\frac{dc(t)}{dt} = -k\,c(t) \tag{18}$$

$$\frac{dc(t)}{c(t)} = -k\,dt \tag{19}$$

In the equations 18 and 19, $c(t)$ refers to the concentration (or the partial pressure) of propane. k refers to the rate coefficient. When the conversion of propane was low, the concentration of propane approximated to the initial value (see the eq. 20).

$$c(t) \approx c(t = 0) \tag{20}$$

As such, the equation 20 can be written as the following.

$$\frac{dc(t)}{c(t = 0)} = -k\,dt \tag{21}$$

The conversion of propane was only related to the reaction time and independent of the partial pressure of propane at the initial reaction stage (eq. 22). Moreover, the equilibrium conversion of propane also varies slightly with the change of propane partial pressure at a low conversion level (Supplementary Fig. 13).

$$\text{Conversion} = -\int_{t=0}^{t} \frac{dc(t)}{c(t=0)} = \int_{t=0}^{t} k\,dt = kt \tag{22}$$

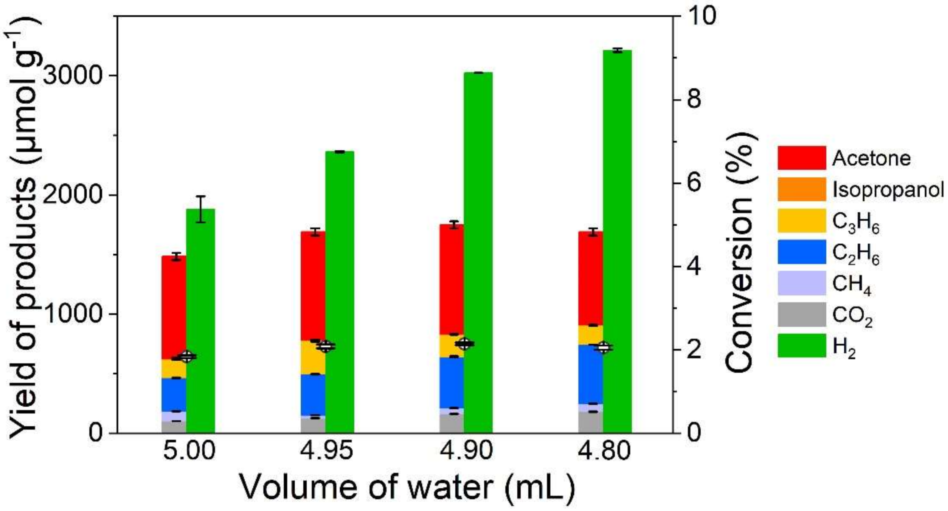

Supplementary Figure 31 | Dependence of products and conversion on the volume of added water. Typically, 25 mg of PtSn/γ-Al_2O_3 (>60 mesh) and different volumes of water were loaded in a 15-mL slurry reactor with the stirring speed of 600 rpm to operate under 6 bar (C_3H_8:N_2 = 5:1) at 350 °C for 2 h. Error bars represent the standard deviation from three independent measurements. Source data are provided as a Source Data file.

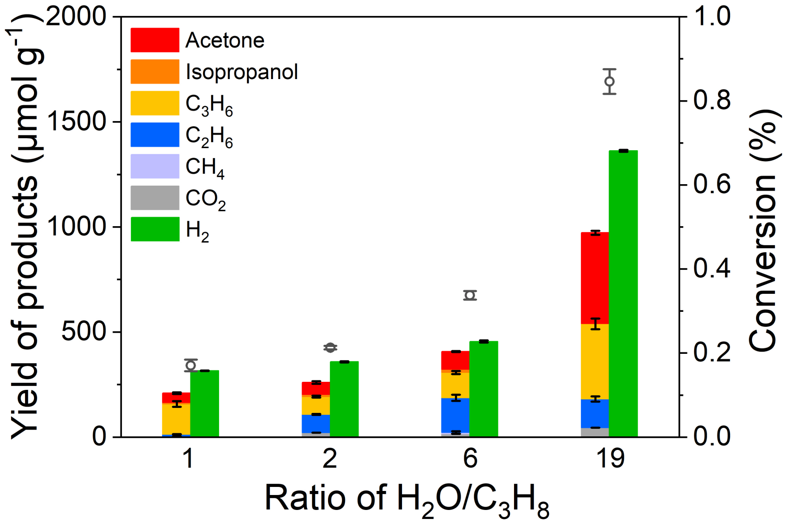

Supplementary Figure 32 | Dependence of product yields on the molar ratio of H_2O/C_3H_8. Typically, 25 mg of PtSn/γ-Al_2O_3 (>60 mesh) and different volumes of water were loaded in a 15-mL slurry reactor with the stirring speed of 600 rpm to operate under 6 bar (C_3H_8:N_2 = 5:1) at 350 °C for 2 h. Error bars represent the standard deviation from three independent measurements. Source data are provided as a Source Data file.

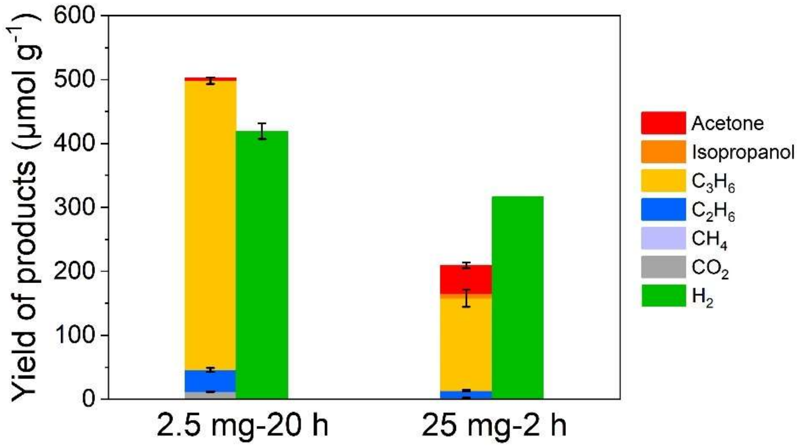

Supplementary Figure 33 | Exploration of the contact between the catalyst and reactants. Typically, PtSn/γ-Al_2O_3 (>60 mesh) and water were loaded in a 15-mL slurry reactor at 350 ºC, in which n(C_3H_8): n(H_2O) = 1. Error bars represent the standard deviation from three independent measurements. Source data are provided as a Source Data file.

Supplementary Table 17 | Dependence of product yields and conversion on the molar ratio of H_2O/C_3H_8.

Entry	H_2O/C_3H_8	V_{H2O} (mL)	$n_{Acetone}$ (μmol g^{-1})	Conversion (%)
1	1	0.05	44.53 ± 4.17	0.17 ± 0.01
2	2	0.10	57.51 ± 6.37	0.21 ± 0.00
3	6	0.30	84.83 ± 2.05	0.34 ± 0.01
4	19	1.00	431.94 ± 9.84	0.85 ± 0.03
5	135	5.00	858.42 ± 30.33	1.84 ± 0.04

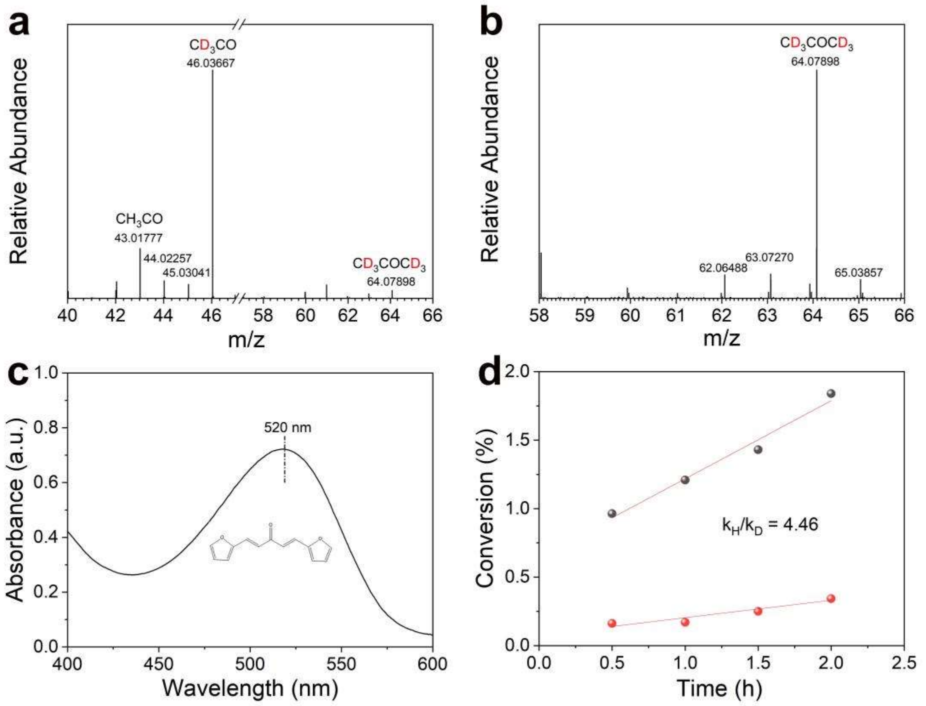

Supplementary Figure 34 | KIE test of propane wet reforming over PtSn/γ-Al_2O_3. (a,b) High resolution mass spectrum of products obtained by propane wet reforming over PtSn/γ-Al_2O_3 in D_2O. (c) UV-vis spectrum of solution obtained through furfural colorimetric method; the inset shows the molecular structure of chromogenic substance. (d) KIE test of propane wet reforming over PtSn/γ-Al_2O_3. Experimental details are provided in Supplementary Note 6. Source data are provided as a Source Data file.

Supplementary Note 6. Experimental details for KIE tests.

The deuterated products were extracted by chloroform and analyzed by the high-resolution mass spectrum. The results were shown in Supplementary Figure 34a and b, indicating that CD_3COCD_3 is the main product. NMR spectrum exhibited no peaks for CD_3COCD_3. Thus, we used the furfural chromogenic method to quantify the acetone (Supplementary Figure 34c). Specifically, 2.5 mL of the solution for test was added to 2.5 mL of NaOH solution (4%), followed by the addition of 1 mL of furfural. After mixing evenly, the solution was heated at 65 ^{o}C for 5 min and quickly cooled in an ice water bath. Afterwards, 2 mL of concentrated H_2SO_4 was slowly added to the solution, resulting in an orange solution for UV-vis test.

Of note, the time zero point was counted when the system temperature reached 350 ^{o}C. However, the reaction had already occurred before the time zero point, which led to a non-zero intercept. The reaction rate was reflected by the slope instead of the intercept and the intercept did not influence the calculation of KIE value.

Supplementary Table 18 | Ratio of peak area propane-pulse experiments in Figure 4a-c.

catalysts	Ratio of peak area			
	$S(C_3H_6)/S(C_3H_8)$	$S(C_2H_6)/S(C_3H_8)$	$S(CH_4)/S(C_3H_8)$	$S(H_2)/S(C_3H_8)$
PtSn/γ-Al_2O_3	0.77	0.11	0.03	1.50
Pt/γ-Al_2O_3	0.62	0.72	0.16	2.46
γ-Al_2O_3	0.05	0.04	0.07	0.05

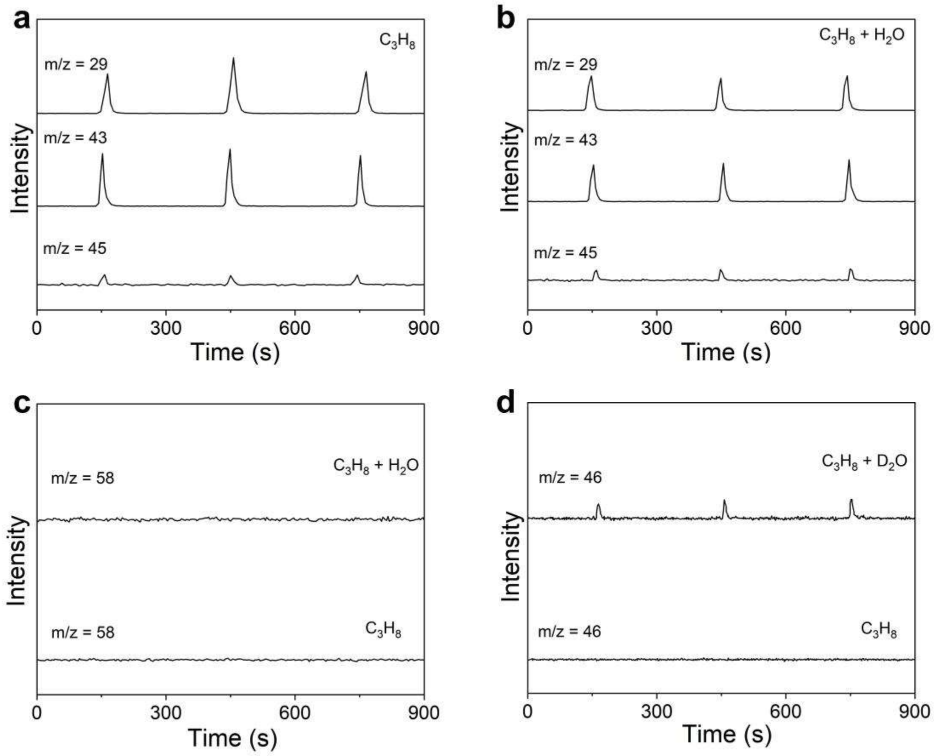

Supplementary Figure 35 | TPSR-MS spectra. (a) Transient response curves of pure propane pulses for the signals at m/z = 29, 43, and 45. (b) Transient response curves of PtSn/γ-Al_2O_3 obtained during wet propane pulses into He flow at 350 °C with the rate of 20 mL min^{-1} for the signals at m/z = 29, 43, and 45. (c) Comparison of pure propane pulses and H_2O/C_3H_8 pulses over PtSn/γ-Al_2O_3 for the signal at m/z = 58. (d) Comparison of pure propane pulses and D_2O/C_3H_8 pulses over PtSn/γ-Al_2O_3 for the signal at m/z = 46. When we pulsed wet propane over PtSn/γ-Al_2O_3, we observed the signals at m/z = 29, 43, and 45. For assignment, the MS profile of pure propane also exhibited the signals at these positions. Considering that the signal at m/z = 43 from acetone was likely overshadowed by that from propane, we turned to the signal at m/z = 58 which was the secondly strongest peak for a typical MS profile of acetone. However, we did not observe this signal during the experiment of pulsing wet propane. In addition, we used propane pulses with D_2O bubble into He flow. The signal at m/z = 46 appeared, implying the formation of isopropanol. Source data are provided as a Source Data file.

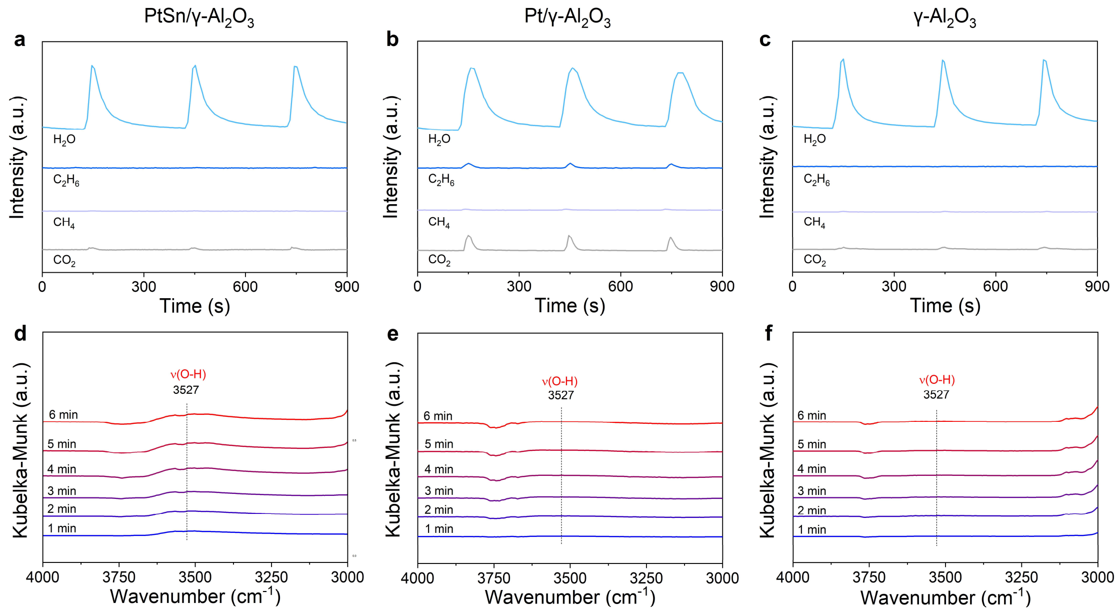

Supplementary Figure 36 | TPSR-MS spectra and in-situ DRIFTS spectra. Transient response curves for C_2H_6, CH_4, and CO_2 obtained during propene pulses with water bubble into He flow over (a) PtSn/γ-Al_2O_3, (b) Pt/γ-Al_2O_3, and (c) γ-Al_2O_3. *In situ* DRIFTS spectra of (d) PtSn/γ-Al_2O_3, (e) Pt/γ-Al_2O_3, and (f) γ-Al_2O_3 obtained during He flow with isopropanol bubble at 350 °C. Source data are provided as a Source Data file.

Supplementary Table 19 | Assignment of DRIFTS peaks for isopropanol in Figure 5a-c.

Surface species	Experimental wavenumber (cm^{-1})	Literature value (cm^{-1})	Reference
ν(O-H)	3527	3420-3667	11, 12
$\nu_{as}(CH_3)$	2970	2975-2963	12
$\nu_{s}(CH_3)$	2887	2880-2878	12
$\delta_{as}(CH_3)$	1474	1472-1465	12
$\delta_{s}(CH_3)$	1381	1389-1359	12
ν(C=O)	1732	1731	13
ν(-COH)	1637	1632	14
ν_{as}(-OCO)	1576	1576-1594	12, 14
δ_{s}(O-H)	1230	1282	12

ν(O-H): the stretch of OH*.
$\nu_{as}(CH_3)$: the symmetric stretch of CH_3*.
$\nu_{s}(CH_3)$: the symmetric stretch of CH_3*.
$\delta_{as}(CH_3)$: the asymmetric bending vibration of CH_3*.
$\delta_{s}(CH_3)$: the symmetric bending vibration of CH_3*.
ν(C=O): the stretch of C=O bonds.
ν(-COH): the stretch of C-O bonds.
ν_{as}(-OCO): the asymmetric stretch of carboxylate.
δ_{s}(O-H): the bending vibration of OH*.

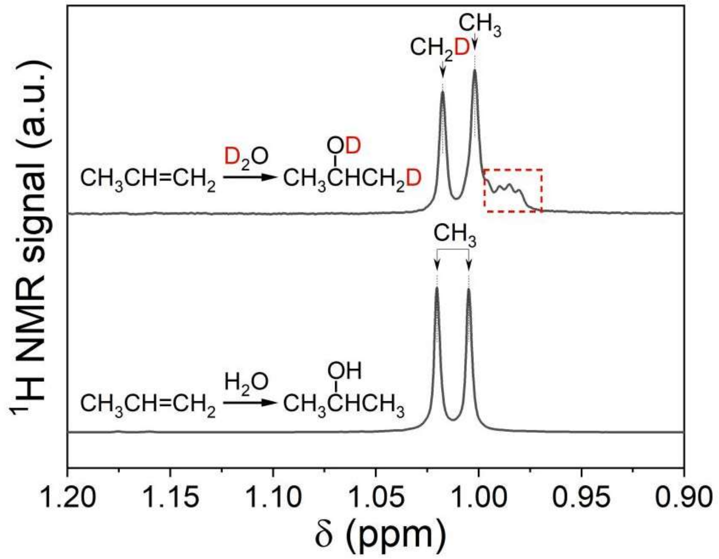

Supplementary Figure 37 | Typical ^{1}H NMR spectra of liquid products obtained by propene hydration over γ-Al_2O_3. Reaction condition: 25 mg of γ-Al_2O_3, 5 mL of water or D_2O, 6 bar (C_3H_6:N_2 = 5:1), 350 ºC, 2 h. Source data are provided as a Source Data file.

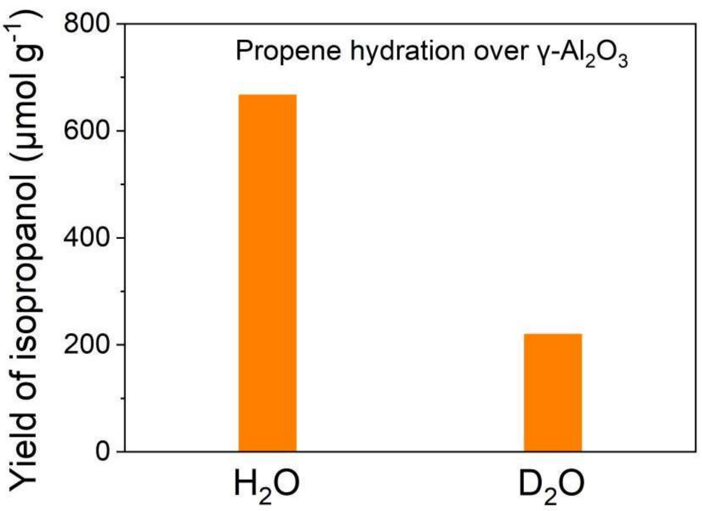

Supplementary Figure 38 | Propene hydration over γ-Al_2O_3 in water or D_2O. Yields of isopropanol obtained in 5 mL of water or D_2O under 6 bar (C_3H_6:N_2 = 5:1) at 350 °C for 2 h over 25 mg of γ-Al_2O_3. Source data are provided as a Source Data file.

Supplementary Table 20 | Possible reactions involved in propene-water system.

$C_3H_6 + H_2O \rightarrow i\text{-}C_3H_7OH$	hydration of propene
$i\text{-}C_3H_7OH \rightarrow (CH_3)_2CO + H_2$	dehydrogenation of isopropanol
$i\text{-}C_3H_7OH + 5H_2O \rightarrow 3CO_2 + 9H_2$	steam reforming of isopropanol
$C_3H_6 + 3H_2O \rightarrow 3CO + 6H_2$	steam reforming of propene
$CO + H_2O \rightarrow CO_2 + H_2$	water-gas shift reaction
$C_3H_6 + H_2 \rightarrow C_3H_8$	hydrogenation of propene

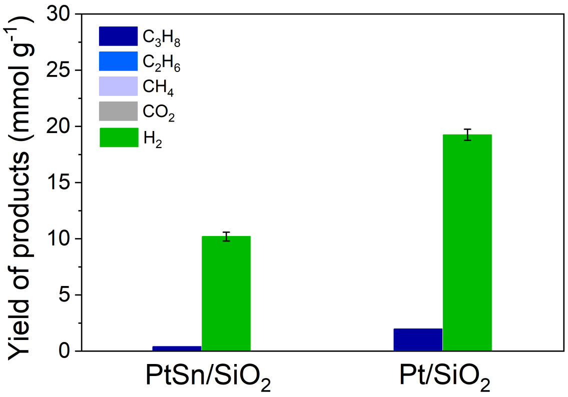

Supplementary Figure 39 | Hydration of propene over PtSn/SiO$_2$ and Pt/SiO$_2$. Typically, 25 mg of the catalyst was operated in 5 mL of water under 6 bar (C_3H_6:N_2 = 5:1) at 350 °C for 2 h. Error bars represent the standard deviation from three independent measurements. Source data are provided as a Source Data file.

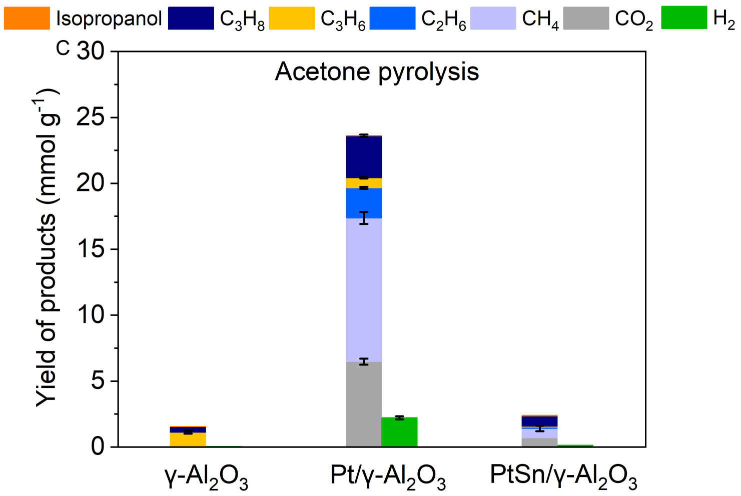

Supplementary Figure 40 | Acetone pyrolysis. Yields of products obtained over 25 mg of catalysts in 4.95 mL of water with 0.05 mL of dissolved acetone under 6 bar N_2 at 350 °C for 2 h. Error bars represent the standard deviation from three independent measurements. Source data are provided as a Source Data file.

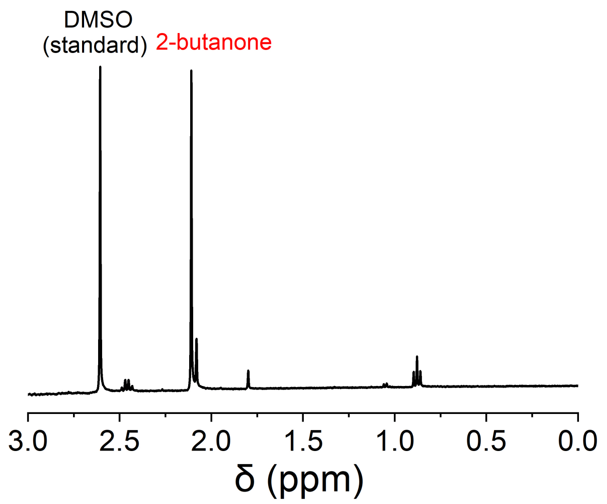

Supplementary Figure 41 | Typical ^{1}H NMR spectrum of liquid products obtained by reaction of water with butane. Reaction condition: 25 mg of PtSn/γ-Al_2O_3, 5 mL of water, 3 bar (C_4H_{10}:N_2 = 2:1), 350 °C, 2 h. DMSO was used as the standard. Source data are provided as a Source Data file.

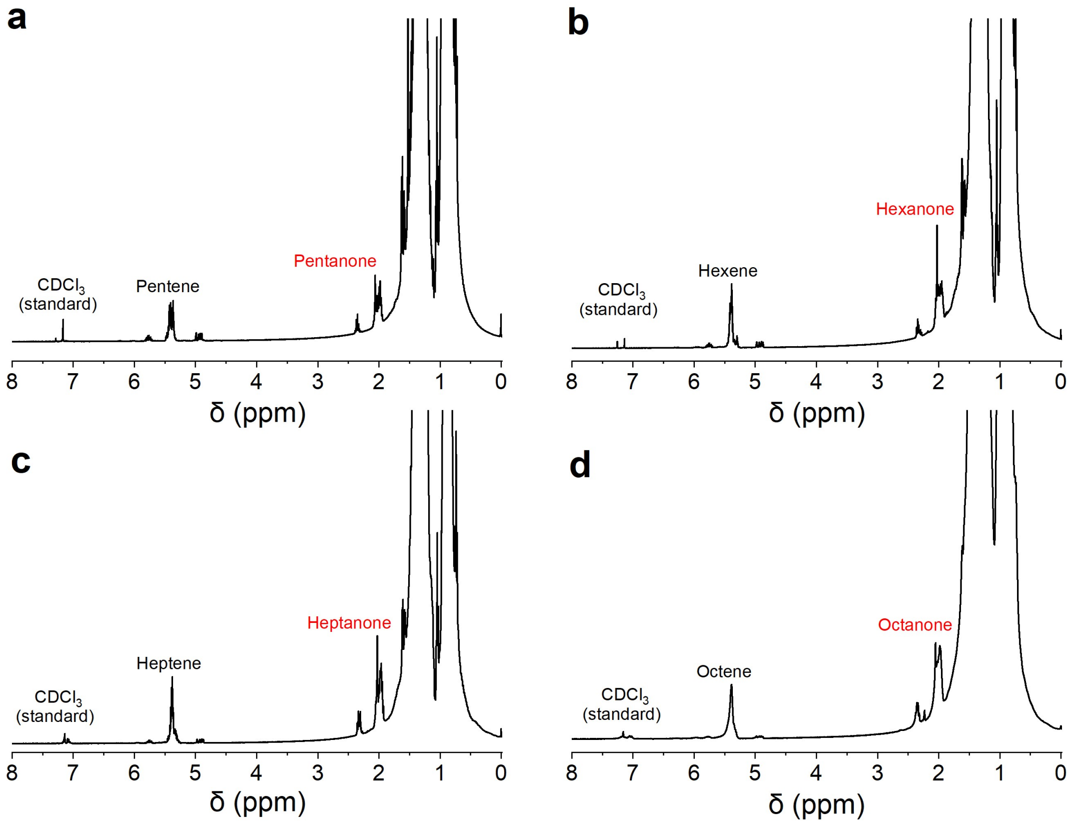

Supplementary Figure 42 | Typical ^{1}H NMR spectra. Typical ^{1}H NMR spectra of liquid products obtained by reaction of water with (a) pentane, (b) hexane, (c) heptane, and (d) octane. Reaction condition: 25 mg of PtSn/γ-Al_2O_3, 2.5 mL of water, 2.5 mL of liquid alkane and 1 bar of N_2, 350 °C, 2 h. $CDCl_3$ was used as the standard. Source data are provided as a Source Data file.

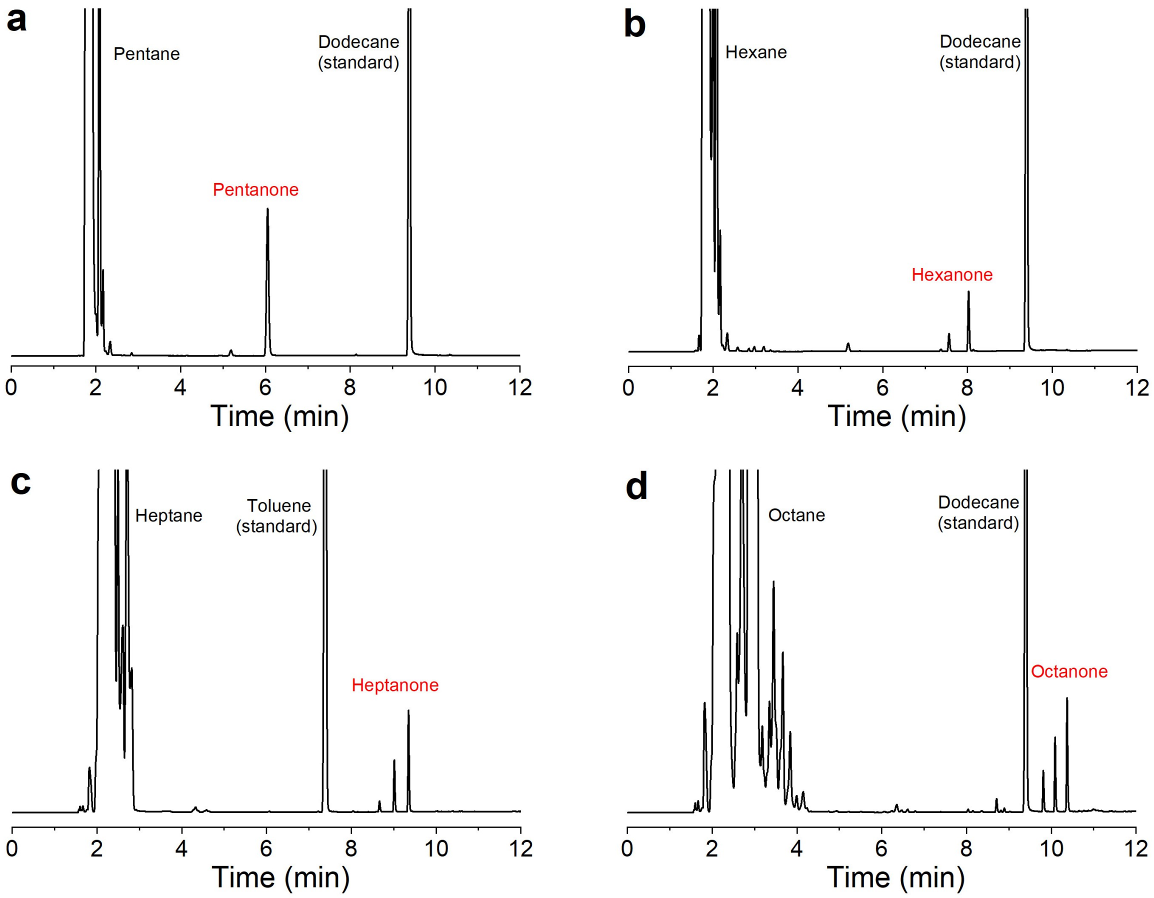

Supplementary Figure 43 | Typical GC spectra. Typical GC spectra of liquid products obtained by reaction of water with (a) pentane, (b) hexane, (c) heptane, and (d) octane. Reaction condition: 25 mg of PtSn/γ-Al_2O_3, 2.5 mL of water, 2.5 mL of liquid alkane and 1 bar of N_2, 350 ºC, 2 h. Dodecane was used as the standard for the quantification of pentanones, hexanones and octanones. Toluene was used as the standard for the quantification of heptanones. Source data are provided as a Source Data file.

Supplementary Table 21 | Catalytic data in wet reforming of butane.

CO_2 (μmol g^{-1})	CH_4 (μmol g^{-1})	C_2H_6 (μmol g^{-1})	C_3H_8 (μmol g^{-1})	olefins (μmol g^{-1})	2-butanone (μmol g^{-1})	H_2 (μmol g^{-1})
416.4	594.0	88.4	164.8	59.6	847.2	1705.2

Supplementary Table 22 | Catalytic data in wet reforming of pentane.

CO_2 (μmol g^{-1})	CH_4 (μmol g^{-1})	C_2H_6 (μmol g^{-1})	C_3H_8 (μmol g^{-1})	olefins (μmol g^{-1})	pentanones (μmol g^{-1}) 2-pentanone	H_2 (μmol g^{-1})
253.6	28.8	6.0	99.2	3528.0	925.6	5826.8

Supplementary Table 23 | Catalytic data in wet reforming of hexane.

CO_2 (µmol g^{-1})	CH_4 (µmol g^{-1})	C_2H_6 (µmol g^{-1})	C_3H_8 (µmol g^{-1})	olefins (µmol g^{-1})	hexanones (µmol g^{-1})		H_2 (µmol g^{-1})
					2-hexanone	3-hexanone	
63.6	16.0	24.8	8.4	3942.0	873.6	267.2	6533.6

Supplementary Table 24 | Catalytic data in wet reforming of heptane.

CO_2 (μmol g^{-1})	CH_4 (μmol g^{-1})	C_2H_6 (μmol g^{-1})	C_3H_8 (μmol g^{-1})	olefins (μmol g^{-1})	heptanones (μmol g^{-1})			H_2 (μmol g^{-1})
					2-heptanone	3-heptanone	4-heptanone	
109.6	18.0	32.0	19.6	4716.8	922.4	492.8	109.2	7255.6

Supplementary Table 25 | Catalytic data in wet reforming of octane.

CO_2 (μmol g^{-1})	CH_4 (μmol g^{-1})	C_2H_6 (μmol g^{-1})	C_3H_8 (μmol g^{-1})	olefins (μmol g^{-1})	octanones (μmol g^{-1})			H_2 (μmol g^{-1})
					2-octanone	3-octanone	4-octanone	
82.0	31.6	67.6	32.8	6580.4	912.8	598.4	337.2	8817.2

Supplementary References

1. Schnohr, C. *et al.* Anisotropic vibrations in crystalline and amorphous InP. *Phys. Rev. B* **79,** 195203 (2009).

2. Fornasini, P. *et al.* On EXAFS Debye-Waller factor and recent advances. *J. Synchrotron Rad.* **22,** 1242–1257 (2015).

3. Yan, H. *et al.* Tandem In_2O_3-Pt/Al_2O_3 catalyst for coupling of propane dehydrogenation to selective H_2 combustion. *Science* **371,** 1257 (2021).

4. Motagamwala, A. *et al*. Stable and selective catalysts for propane dehydrogenation operating at thermodynamic limit. *Science* **373,** 217 (2021).

5. Avanesian, T. *et al*. Quantitative and atomic-scale view of CO-induced Pt nanoparticle surface reconstruction at saturation coverage via DFT calculations coupled with in situ TEM and IR. *J. Am. Chem. Soc.* **139,** 4551–4558 (2017).

6. Wang, P. *et al*. Stabilizing the isolated Pt sites on PtGa/Al_2O_3 catalyst via silica coating layers for propane dehydrogenation at low temperature. *Appl. Catal. B: Environ.* **300,** 120731 (2022).

7. Zhang, W. *et al.* Size dependence of Pt catalysts for propane dehydrogenation: from atomically dispersed to nanoparticles. *ACS Catal.* **10**, 12932-12942 (2020).

8. Allian, A. *et al.* Chemisorption of CO and mechanism of CO oxidation on supported platinum nanoclusters. *J. Am. Chem. Soc.* **133**, 4498–4517(2011).

9. Zhang, M. *et al.* How to measure the reaction performance of heterogeneous catalytic reactions reliably. *Joule* **3**, 2876-2883 (2019).

10. Wang, H. *et al.* Coke formation on Pt–Sn/Al_2O_3 catalyst for propane dehydrogenation. *Ind. Eng. Chem. Res.* **57**, 8647-8654 (2018).

11. Guo, M. *et al*. Synergy in Au-CuO Janus structure for catalytic isopropanol oxidative dehydrogenation to acetone. *Angew. Chem. Int. Ed.* **61,** e202203827 (2022).

12. Zhang, H. *et al*. A Resource utilization method for volatile organic compounds emission from the semiconductor industry: Selective catalytic oxidation of isopropanol to acetone over Au/α-Fe_2O_3 nanosheets. *Appl. Catal. B: Environ. Energy* **175,** 119011 (2020).

13. Luo, L. *et al*. Selective photoelectrocatalytic glycerol oxidation to dihydroxyacetone via enhanced middle hydroxyl adsorption over a Bi_2O_3-incorporated catalyst. *J. Am. Chem. Soc.* **144,** 7720–7730 (2022).

14. Jiang, Z. *et al*. Modulating the electronic metal-support interactions in single-atom Pt_1-CuO catalyst for boosting acetone oxidation. *Angew. Chem. Int. Ed.* **61,** e202200763 (2022).
